# Long-term caffeine treatment of Alzheimer mouse models ameliorates behavioural deficits and neuron loss and promotes cellular and molecular markers of neurogenesis

**DOI:** 10.1007/s00018-021-04062-8

**Published:** 2021-12-16

**Authors:** Martina Stazi, Sandra Lehmann, M. Sadman Sakib, Tonatiuh Pena-Centeno, Luca Büschgens, Andre Fischer, Sascha Weggen, Oliver Wirths

**Affiliations:** 1 Department of Psychiatry and Psychotherapy, University Medical Center (UMG), Georg-August-University, Von-Siebold-Str. 5, 37075 Göttingen, Germany; 2 Department of Neuropathology, Heinrich-Heine-University, Düsseldorf, Germany; 3 German Center for Neurodegenerative Diseases (DZNE), Göttingen, Germany

**Keywords:** Alzheimer’s disease, Transgenic mice, Amyloid, Caffeine, Behavior, Neurogenesis, Transcriptome, RNA sequencing, 5xFAD, Tg4-42

## Abstract

Epidemiological studies indicate that the consumption of caffeine, the most commonly ingested psychoactive substance found in coffee, tea or soft drinks, reduces the risk of developing Alzheimer’s disease (AD). Previous treatment studies with transgenic AD mouse models reported a reduced amyloid plaque load and an amelioration of behavioral deficits. It has been further shown that moderate doses of caffeine have the potential to attenuate the health burden in preclinical mouse models of a variety of brain disorders (reviewed in Cunha in J Neurochem 139:1019–1055, 2016). In the current study, we assessed whether long-term caffeine consumption affected hippocampal neuron loss and associated behavioral deficits in the Tg4-42 mouse model of AD. Treatment over a 4-month period reduced hippocampal neuron loss, rescued learning and memory deficits, and ameliorated impaired neurogenesis. Neuron-specific RNA sequencing analysis in the hippocampus revealed an altered expression profile distinguished by the up-regulation of genes linked to synaptic function and processes, and to neural progenitor proliferation. Treatment of 5xFAD mice, which develop prominent amyloid pathology, with the same paradigm also rescued behavioral deficits but did not affect extracellular amyloid-β (Aβ) levels or amyloid precursor protein (APP) processing. These findings challenge previous assumptions that caffeine is anti-amyloidogenic and indicate that the promotion of neurogenesis might play a role in its beneficial effects.

**Supplementary Information:**

The online version contains supplementary material available at 10.1007/s00018-021-04062-8.

## Introduction

Epidemiological studies had suggested that coffee intake might be inversely linked with a variety of different diseases, such as type 2 diabetes [2], cardiovascular [3] or neurodegenerative diseases [4]. In particular, several human studies had indicated that a daily caffeine consumption equivalent to 3 or more cups of coffee reduced cognitive decline in woman and man without dementia [5–7]. In a case–control study, plasma caffeine levels were found to be significantly lower in mild cognitive impairment (MCI) subjects that later converted to dementia compared to stable MCI subjects [8]. Other studies had focused on possible beneficial effects of caffeine in patients suffering from Alzheimer’s disease (AD). It has been reported that plasma and cerebrospinal fluid (CSF) levels of the caffeine metabolite theobromine showed a significant positive correlation with CSF Aβ42 levels, though no correlation between caffeine consumption and CSF Aβ42 levels was established [9]. A recent study from South Korea described that a lifetime intake of 2 or more cups of coffee per day is associated with lower brain Aβ positivity, as assessed by Pittsburgh compound B positron emission tomography (PET) [10]. In a 21-year follow-up study, the consumption of 3–5 cups of coffee starting in midlife was associated with a reduced risk of AD in later life [11]. In another study, AD patients were found to have consumed considerably less caffeine during the 20 years preceding their AD diagnosis when compared to age-matched non-AD patients [12]. Although these correlative findings suggest that long-term caffeine consumption may protect against cognitive decline, important caveats are that these retrospective studies are based only on the memorization of the patients, and that the effects of caffeine might be influenced by confounders such as personal lifestyle choices (e.g. diet or physical activity). However, experimental studies in different preclinical rodent models of dementia have also provided evidence that oral caffeine intake might be able to mitigate cognitive impairment [13–16]. While it has been demonstrated that the effects of caffeine on synaptic transmission and hippocampal plasticity are mainly mediated by a selective antagonism of adenosine receptors [1, 17, 18], there is also evidence that caffeine might act potentially through direct suppression of brain Aβ production [19–21]. However, it has to be noted that caffeine also prevents memory dysfunction in numerous conditions unrelated to amyloid production [1], such as attention-deficit hyperactivity disorder [22], diabetic encephalopathy [23], convulsions [24], chronic stress [18] or depression [25].

Here, we evaluated the effects of chronic oral caffeine intake in two transgenic AD mouse models, 5xFAD and Tg4-42, providing a wider spectrum of AD phenotypes including progressive neuronal loss. Tg4-42 mice overexpress the Aβ_4-42_ peptide sequence, one of the most abundant Aβ variants in human AD brain [26], but without concomitant overexpression of the human amyloid precursor protein (APP) and without any mutations linked to autosomal-dominant forms of AD. These mice present with age-dependent CA1 neuronal loss, accompanied by memory and motor deficits and impaired hippocampal neurogenesis [27–29]. The widely used and well-characterized 5xFAD mouse model overexpresses mutant forms of human APP and presenilin-1 (PSEN1) under the control of the murine Thy1 promoter [30]. These animals develop typical AD hallmarks including robust extracellular amyloid plaque deposition, working memory impairment, and neuroinflammation in an age-dependent manner [30–32]. We report that long-term oral caffeine intake was able to completely rescue the observed learning and memory deficits in both of the AD mouse models. Moreover, caffeine supplementation reduced the CA1 neuronal loss and ameliorated impaired neurogenesis in the Tg4-42 mouse model. Strikingly, the extracellular Aβ plaque load, brain Aβ_1–42_ levels, and the neuroinflammatory phenotype of 5xFAD mice were largely unaffected by caffeine treatment.

## Materials and methods

### Mice

Generation of the Tg4-42 mouse model has been described previously [33]. In brief, this mouse model uses the murine Thy1 promotor to overexpress a genetic construct comprising the human Aβ_4-42_ sequence fused to the murine thyrotropin-releasing hormone (TRH) signal peptide, promoting Aβ_4-42_ secretion. Tg4-42 mice were generated and maintained in a homozygous manner on a C57Bl/6 J genetic background. 5xFAD mice (line Tg6799) mice [30] have been back-crossed for more than ten generations to C57BL/6 J wild type mice and were maintained on a C57BL/6 J genetic background as a heterozygous transgenic line. These mice overexpress human APP695 (carrying the Swedish, Florida and London mutations), as well as mutant human presenilin-1 (PSEN-1), (with the M146L and L286V mutations), both under the control of the murine Thy1 promoter. C57Bl/6 J mice (WT) served as controls (Jackson Laboratories, Bar Harbor, ME, USA). In this study, both male and female animals were used. All animals were handled according to German guidelines for animal care and all experiments have been approved by the local animal care and use committee (Landesamt für Verbraucherschutz und Lebensmittelsicherheit (LAVES), Lower Saxony).

### Treatments

**Fig. 1:**
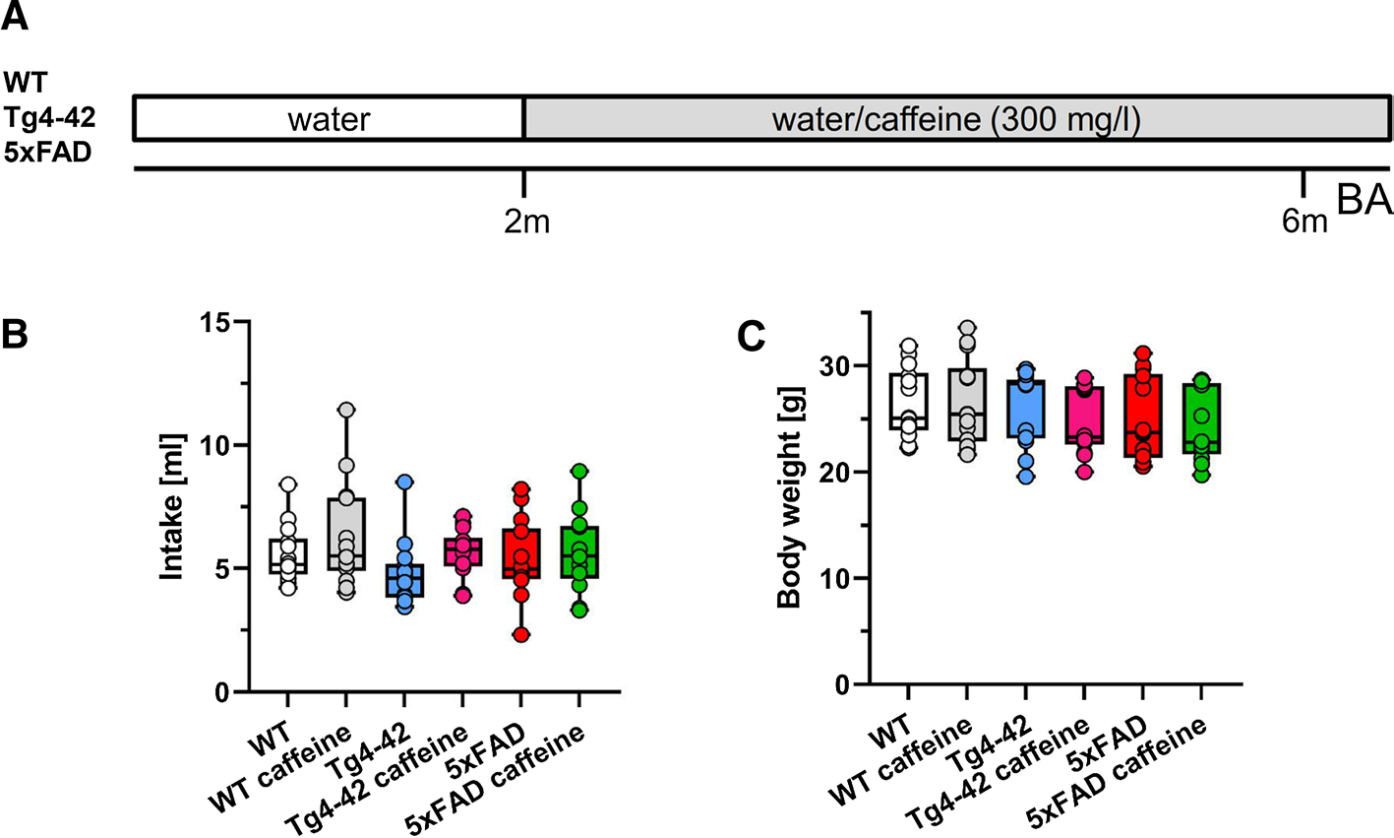
Experimental design and housing conditions. **A** At 2 months of age, WT, 5xFAD or Tg4-42 mice received either tap water or water supplemented with caffeine (300 mg/l) for 4 months. At the end of the treatment period, behavioral performance and various parameters of brain pathology were assessed. No differences in daily water consumption **B** or body weight **C** were detected among the different experimental groups during the behavioral testing (*n* = 14 per group). One-way ANOVA with Tukey’s multiple comparison tests. Data are given as means ± SD. *BA* behavioural analysis

Chronic oral caffeine treatment was initiated at 2 months of age (Fig. 1A). Caffeine (Sigma-Aldrich, #C0750) was administered orally via drinking water at a dose of 300 mg/l [20, 34] for a period of 4 months and was maintained until mice were sacrificed [24]. Water consumption was measured daily during the behavioural analysis to assess average daily caffeine intake in all treatment groups, and in the control groups (WT, Tg4-42^hom^ and 5xFAD) receiving tap drinking water. Mice were kept in groups of 3–4 animals in standard cages equipped with cardboard roles and nesting material. Access to food and water was provided ad libitum.

### Behavioural testing

To assess potential beneficial effects of long-term caffeine treatment with regard to learning and motor behaviour, Tg4-42 and 5xFAD mice were tested at the end of the treatment period at 6 months of age in a set of anxiety, motor, as well as learning and memory tests [WT, WT caffeine, Tg4-42, Tg4-42 caffeine, 5xFAD, 5xFAD caffeine (*n* = 13–14 each)]. Animals were kept on a 12 h/12 h inverted light/dark cycle (light phase from 8 PM to 8 AM) and were sacrificed immediately after the last day of testing. All behaviour experiments were carried out during the dark phase between 9.00 AM and 3 PM. A scheme illustrating the behavioural testing timeline is shown in Supplementary Fig. 1.

### Accelerating rotarod

Motor coordination and balance skills were analysed using the accelerating rotarod test [35] (RotaRod, TSE Systems GmbH, Bad Homburg, Germany). Over two consecutive days (4 session per day with 15 min inter-trial interval), each mouse was individually placed on the rod [acceleration from 4 to 40 revolutions per minute (rpm)] over a maximum session duration of 5 min. Sessions were finished when animals fell off, or the maximum time was reached, and latency to fall (s) was recorded as an indicator of motor abilities. The apparatus was cleaned between sessions with 70% ethanol solution to avoid odour cues.

### Balance beam

The balance beam was used to evaluate fine motor coordination and balance of the mice as described previously [31]. A beam (50 cm × 1 cm) was attached between two platforms (9 cm × 15 cm), elevated 45 cm above a cushioned surface. Animals were placed at the centre of the beam, facing one of the two platforms and the latency to fall was recorded as the average of three 1 min trials. Mice were allowed to rest for at least 10 min between trials. If the mouse managed to reach a platform or remained on the beam throughout the trial, the maximum time (60 s) was recorded. Between the trials, the apparatus was cleaned with 70% ethanol solution to diminish odour cues.

### Elevated plus maze

The elevated plus maze (EPM) was used to assess exploratory behavior and anxiety levels [31]. The EPM is raised 75 cm above a padded surface and consists of four arms in a “ + ” configuration of 15-cm length and 5-cm width. Two oppositely positioned arms contained lateral walls (closed arms), whereas the other pair of arms were opened (open arms). Experiments were carried out under red light conditions, and mice were placed in the central area of the apparatus facing one of the open arms. Mice were allowed to explore the maze freely for 5 min. Travelled distance, arm entries, average speed and time percentage spent in each arm were recorded and calculated using the ANY-Maze tracking software (Stoelting Europe). Anxiety-like behavior was calculated using the time spent in the open arms, with longer times spent in the open arms corresponding to reduced anxiety levels [36]. To eliminate odor cues, the EPM was cleaned after each mouse using 70% ethanol.

### Open field, novel object recognition and novel object location

The open field (OF) test was used to analyze locomotor activity, exploratory behaviour and anxiety levels. During the OF test, mice were placed in the middle of a square box (50 × 50 cm), which they could freely explore for 5 min. The total time spent in the central part of the arena, as well as the total distance travelled, and the average speed were recorded using a video-tracking software (ANY-maze, Stoelting Europe).

Twenty-four hours after the OF, the novel object recognition (NOR) or the novel object location (NOL) tests were performed in the same box, now containing two identical objects (training phase). The NOR is a commonly used behavioural task to evaluate in particular recognition memory and novelty preference [37], and it has been previously used with Tg4-42 mice [28]. Mice were allowed to freely explore the objects for 5 min. Twenty-four hours later, one of the two objects was replaced with a novel one consistent in height and volume but different in shape and appearance (testing phase).

We did not observe deficits in 12-month-old 5xFAD mice with the NOR in a previous study [38], which is in line with data for 6-month-old 5xFAD mice published by others [39, 40], while the NOL task has been successfully used in 5xFAD mice at this time point [41]. The NOL memory task evaluates spatial memory and is based on the ability of mice to recognize when a familiar object has been relocated. Twenty-four h after the OF, during the training phase, 5xFAD mice could freely explore two duplicate objects (O_1_ and O_2_), which were placed close to the far corners of the arena for 5 min. After a delay of 24 h, one object was placed in the diagonally opposite corner. Thus, in the testing phase, both objects were equally familiar but one was now in a new location [41, 42].

For both of the memory tests, object exploration was scored whenever the mouse sniffed the objects while looking at them [43]. Data collection and video analysis were performed blind to the genotype and the experimental conditions. The percentage of exploration time for the novel object/novel location and discrimination indices (DI) was calculated as follows:

$$\begin{aligned}& \% {\text{ exploration time}}\,\, = \,\,\frac{{{\text{time}}\,{\text{at}}\,{\text{novel}} \times 100}}{{{\text{total}}\,{\text{exploration}}\,{\text{time}}}}\,\,\,\,{\text{and}}\\&{\text{DI}}\,{ = }\,\frac{{{\text{time}}\,{\text{at}}\,{\text{novel - time}}\,{\text{at}}\,{\text{familiar}}}}{{{\text{total}}\,{\text{exploration}}\,{\text{time}}}} \end{aligned}$$

In between trials, the arenas as well as the objects were cleaned with 70% ethanol to diminish odour cues.

### Morris water maze

The Morris Water Maze test (MWM) [44] was used to assess spatial reference memory as previously described. In brief, mice were trained to learn to localize a submerged platform (ø 10 cm) in a circular pool (ø 110 cm) filled with water made opaque with non-toxic white paint. This test consisted of two learning phases and one final testing phase. First, a “cued training” was carried out on 3 consecutive days (4 trials per day, each one for a maximum of 1 min), in which the submerged platform was marked with a triangular visible flag. Twenty-four h after the last trial of the cued training, mice performed 5 days of “acquisition training” (4 trials per day, each one for a maximum of 1 min) in which proximal cues were added around the pool and the flag was removed from the platform. Another 24 h later, a “probe trial” was used to assess spatial reference memory. During this one-minute trial, proximal and distal cues remained in the same position, but the platform was removed. Since the platform location was kept constant during the acquisition training, mice with intact spatial reference memory exhibit a target quadrant preference. Between the trials, mice were dried and kept under infrared light to prevent hypothermia. All trials were recorded using a video-tracking software (ANY-maze, Stoelting Europe) and parameters, such as escape latency, swimming speed, swimming path quadrant preference, latency to first entry into the platform/target quadrant, time into the platform/target quadrant, and entries into the platform/target quadrant were recorded.

### Tissue collection and preservation

Mice were sacrificed the day after finishing the behavioural experiments between 9.00 and 12.00 a.m. In brief, mice were deeply anesthetized and transcardially perfused using ice-cold 0.01 M Phosphate-buffered saline (PBS), and brains were removed and carefully dissected. Tissues were collected depending on the application as follows: for the Tg4-42 female mice, the right hemisphere (RH) was post-fixed in 4% formalin solution at 4 °C for at least 72 h, protected from the light, before embedding in paraffin. The left hemisphere (LH) of the Tg4-42 female mice was post-fixed in 4% paraformaldehyde (PFA) in 0.01 M PBS for at least 24 h before being transferred to a 30% sucrose solution (in 0.01 PBS) for cryo-protection. Finally, brain tissue was deep-frozen on dry ice and stored at − 80 °C until further use. For the Tg4-42 male mice, entire hippocampi and cortices from both hemispheres were carefully dissected, deep-frozen on dry ice and stored at − 80 °C until further use. For the 5xFAD mice, the RH was collected as described for the Tg4-42 female mice, and the LH was dissected as described for the Tg4-42 male mice.

### Quantification of CA1 neuron numbers

Neuronal quantification in the CA1 layer of the hippocampus was performed with 4 μm sagittal paraffin brain sections (Bregma 1.08–1.32 according to [45]) cut on a rotation microtome (Microm, HM335E, Thermo Fisher Scientific, Germany). The slices were collected in a standardized fashion and stained with haematoxylin [46]. Neuronal nuclei were determined by their size and distinctive characteristics clearly differentiating them from glial cells. Images of the CA1 area of the hippocampus were acquired at × 400 magnification using an Olympus BX-51 microscope equipped with a Moticam pro 282 camera (Motic, Wetzlar, Germany). The number of CA1 neurons per section (*n* = 6 per group, 3 sections per animal, at least 30 µm apart) was counted using the manual cell counting tool implemented in ImageJ (version 1.52u, NIH) and calculated as relative results with the untreated WT group as a reference (100%). The experimenter was blinded with regard to genotype and treatment throughout the analysis.

### Analysis of adult neurogenesis

Frozen cryo-protected brain hemispheres were cut into a series of 30 μm thick coronal sections using a cryostat (CM1850 UV, Leica, Germany). Every 10th coronal frozen section was stained using a free-floating staining protocol to quantify the number of new-born neurons. First, brain sections series were rehydrated for 10 min with ice cold 0.01 M PBS and endogenous peroxidase activity was blocked for 30 min with 0.3% H_2_O_2_ in 0.01 M PBS. Sections were washed in PBS-T (0.01% Triton X-100) for membrane permeabilization. Blocking of nonspecific bindings sites was performed for 1 h by incubation in 0.01 M PBS containing 10% fetal calf serum (FCS) and 4% milk powder at room temperature (RT). The primary goat antibody against doublecortin (DCX, sc-8066, 1:500, Santa Cruz Biotechnology, RRID:AB_2088494) was diluted in 0.01 M PBS containing 10% FCS and incubated overnight at RT. The quantification of DCX-expressing cells has been demonstrated to allow for an accurate measurement of modulations in the rate of adult neurogenesis [47]. On the next day, sections were thoroughly washed with PBS-T and incubated with a secondary anti-goat biotinylated antibody (DAKO, Glostrup, Denmark). Staining was visualized using the ABC method using a Vectastain kit (Vector Laboratories, Burlingame, USA) and diaminobenzidine (DAB) as a chromogen. The total number of newborn neurons was counted in the dentate gyrus (DG) using the meander scan option of StereoInvestigator 7 (MicroBrightField, Williston, USA) to quantify all DCX-positive cells in a given section. The resulting neuron numbers were multiplied by 10 to calculate the total number of newborn neurons per hemisphere. The experimenter was blinded to genotype and treatment throughout the entire analysis. To avoid possible bias due to gender-dependent differences in brain size, only female mice were used for the quantification of CA1 neuron numbers and adult neurogenesis (*n* = 6 per group).

### Immunohistochemistry on paraffin sections

For immunohistochemistry, 4 µm sections were deparaffinized in xylene for 10 min and rehydrated by washes with decreasing ethanol concentrations (100%, 95% and 70% EtOH). After treatment with 0.3% H_2_O_2_ in 0.01 M PBS for 30 min to block endogenous peroxidases, antigen retrieval was performed by boiling sections in 0.01 M citrate buffer pH 6.0, followed by permeabilization in 0.01 M PBS incl. 0.1% Triton X-100 and 3 min treatment with 88% formic acid. Non-specific-binding sites were blocked for one hour at RT by treatment with milk and fetal calf serum in PBS prior to the addition of the primary antibodies. The following primary antibodies were used: Aβ_1-x_ (mouse monoclonal, clone 82E1, 1:1000, Cat. No. JP10323, IBL International, RRID:AB_10707424 [48]), 24311 (rabbit polyclonal, pan-Aβ, 1:500, [48, 49]), glial fibrillary acidic protein (GFAP) (rabbit polyclonal, 1:1000, Cat. No.173002, Synaptic Systems, RRID:AB_887720) and Desmoplakin (rabbit polyclonal, 1:250, Cat. No. NBP2-48836, Novus Biologicals). Primary antibodies were diluted in 10% fetal calf serum in 0.01 M PBS and incubated overnight at RT, followed by incubation with biotinylated anti-mouse and anti-rabbit secondary antibodies (Dianova, 1:200) for 1 h at 37 °C. Staining was visualized via the ABC method using a Vectastain kit (Vector Laboratories, Burlingame, CA, USA) and DAB.

### Quantification of Aβ plaque load and GFAP immunoreactivity

The extracellular Aβ plaque load was evaluated with anti-Aβ antibodies 24311 and 82E1 [48] in the cortex (Co), dentate gyrus (DG), subiculum (Subi), and thalamus (Thal) using an Olympus BX-51 microscope equipped with a Moticam Pro 282A camera (Motic) and the ImageJ software package (V1.41, NIH, USA) as described previously. Images of × 100 magnification were captured on 3 tissue sections per mouse, which were at least 30 μm apart from each other. Using ImageJ, pictures were binarized to 8-bit black and white images, and a fixed intensity threshold was applied defining the DAB signal. Measurements were performed for the percentage area covered by DAB [50]. Similarly, for GFAP staining quantification, images of × 200 magnification were captured and the astrocyte-covered areas were analysed as described previously [49]. The relative Aβ plaque load or GFAP immunoreactivity is expressed normalized to untreated 5xFAD mice.

### ELISA and western blotting

Frozen hippocampi and cortices of 6-month-old 5xFAD mice were homogenized in 700 μl of Tris-buffered saline (TBS) buffer (120 mM NaCl, 50 mM Tris, pH 8.0 with complete protease inhibitor cocktail, Roche) per 100 mg tissue using a Dounce homogenizer (800 rpm). The resulting solution was centrifuged at 17,000×*g* for 20 min at 4 °C. The supernatant containing TBS-soluble proteins was stored at − 80 °C. The pellet was dissolved in 800 μl (cortex) or 200 μl (hippocampus) of 2% SDS and sonicated followed by a centrifugation step at 17,000×*g* for 20 min at 4 °C. The supernatant with SDS-soluble proteins was transferred to a new tube and incubated with 1 μl of benzonase at RT for 10 min followed by storage at − 80 °C.

APP processing was analysed by Western blotting in TBS- and SDS-soluble hippocampal brain fractions of untreated and caffeine-treated 5xFAD mice. Total protein concentrations were determined with a BCA Protein Assay Kit (ThermoFisher Scientific), and equal amounts of protein were separated on 12% Bis–Tris SDS-PAGE gels and transferred onto PVDF membranes (Merck) by electroblotting. The membranes were blocked with 5% milk powder in TBST (25 mM Tris, 137 mM NaCl, 2.7 mM KCl, 0.1% Tween 20, pH 7.4) for 1 h at RT, and then incubated overnight at 4 °C with the primary antibody diluted in TBST. The following primary antibodies against APP were used: CT-15 (rabbit polyclonal raised against the C-terminal 15 amino acids of human APP, 1:3500) [51]; 22C11 (mouse monoclonal raised against residues 66–81 of human APP, 1:1000, kindly provided by Dr. Stefan Kins, University of Kaiserslautern, Germany) [51]; anti-APPs-α (mouse monoclonal, clone 2B3, recognizing the C-terminal neoepitope generated by α-secretase cleavage of APP, 1:50, IBL Cat. No. 11088); anti-Actin (rabbit polyclonal, 1:2000, Sigma-Aldrich Cat. No. A2066); anti-Tubulin (mouse monoclonal, clone DM1A, 1:5000, Sigma-Aldrich Cat. No. T6199). Subsequently, a secondary antibody labelled with a near-infrared fluorescent dye (IRDye 800CW goat anti-mouse IgG or goat anti-rabbit IgG, LI-COR Biosciences) diluted in TBST was added and incubated for 1 h at RT. Fluorescence signals were detected with the Odyssey CLx Imaging System and quantified using the Image Studio Software 2.1 (LI-COR Biosciences).

Aβ_1-42_ peptide levels were determined in the SDS-fractions of cortex and hippocampal homogenates using a sandwich enzyme-linked immunosorbent assay (ELISA) as described with minor modifications [51]. To detect full-length Aβ1–42 peptides, monoclonal antibody IC16 [52] was used as a capture antibody and combined with Aβ_42_ C-terminus-specific detection antibody BAP15 [53]. A standard curve was generated with synthetic Aβ_1-42_ peptides (JPT). 96-well high-binding microtiter plates (Greiner Bio-One) were incubated overnight at 4 °C with the capture antibody in PBS, pH 7.2. After excess capture antibody was removed, freshly diluted brain samples or Aβ_1-42_ peptide standards (in PBS, 0.05% Tween 20, 0.5% BSA) were added. Then, the detection antibody labelled with horseradish peroxidase using the Pierce EZ-Link Plus Activated Peroxidase kit (ThermoFisher Scientific) and diluted in PBS, 0.05% Tween 20, 0.5% BSA was added to each well and incubated overnight at 4 °C. Plates were washed three times with PBS containing 0.05% Tween 20 and once with PBS. Subsequently, 50 μl of trimethylbenzidine ELISA peroxidase substrate (ThermoFisher Scientific) was added and incubated for 1–5 min at RT in the dark. The reaction was terminated by adding 50 μl of 2 M H_2_SO_4_, and the absorbance was recorded using a Paradigm microplate reader (Beckman Coulter) at 450 nm.

### Neuron-specific nuclear RNA sequencing

Nuclei isolation from frozen mouse hippocampal tissues (*n* = 3 per group) was performed according to a previously published protocol [54] with modifications. Isolated nuclei were stained with Anti-NeuN-Alexa488 conjugated antibody (Milipore, MAB377) for 1 h at 4 °C and neuronal nuclei were sorted using BD FACS Aria III sorter based on NeuN signal. Sorted nuclei were collected and RNA was isolated using Trizol LS reagent (Invitrogen) and Zymo RNA clean & concentrator-5 kit (Zymo R1014). RNAseq libraries were prepared using SMART-Seq v4 Ultra Low Input RNA Kit (Takara), followed by Nextera XT library preparation kit (Illumina) according to the manufacturers’ guidelines. Libraries were sequenced in Illumina Hiseq 2000 to get single end 50-base-pair reads. Raw reads were mapped to the mouse genome (mm10) using *STAR* aligner and gene-exon counts were obtained by *subread-featurecounts*. Differential expression analysis was performed using DESeq2. GO-term analysis was performed using Gene Ontology (http://geneontology.org/) and ShinyGO v0.60 [55]. Gene expression data have been deposited in the Gene Expression Omnibus (GEO) database under accession number GSE183323.

### Statistical analysis

Differences between groups were tested with either one-way or two-way analysis of variance (ANOVA) followed by Tukey’s multiple comparison tests or unpaired t test as indicated. Significance levels were defined as follows: ***p < 0.001; **p < 0.01; *p < 0.05. All statistics were calculated using GraphPad Prism version 8.4 for Windows (GraphPad Software, San Diego, CA, USA). Estimation statistics were done as described in [56]. In brief, for estimation based on confidence intervals (CIs), raw data were directly introduced in https://www.estimationstats.com/ [57] and the results and graphs were downloaded.

## Results

### Caffeine does not affect water consumption or body weight of WT, Tg4-42 and 5xFAD mice.

Starting at 2 months of age, WT, 5xFAD or Tg4-42 mice received either tap water or water supplemented with caffeine (300 mg/l) for a period of 4 months (Fig. 1A). Among the different experimental groups, no differences in water consumption were detected (Fig. 1B). In addition, body weight was recorded during the behavioural testing period and no significant differences were detected between the groups irrespective of the treatment (Fig. 1C).

### Limited caffeine effects on motor performance and anxiety in Tg4-42 and 5xFAD mice

Motor performance using the rotarod, balance beam and inverted grid tasks were analysed after 4 months of caffeine treatment. At this time point, no differences were noted between treated and untreated WT (Supplementary Fig. 2A), Tg4-42 (Supplementary Fig. 2B) and 5xFAD mice (Supplementary Fig. 2C). However, Tg4-42 mice performed worse than WT control mice. This was also true for performance in the balance beam task (*p* < 0.001), but caffeine treatment partially rescued this phenotype resulting in a significantly improved performance of Tg4-42 mice (*p* < 0.01; Supplementary Fig. 2D). Both Tg4-42 mice (*p* < 0.05) and 5xFAD mice (*p* < 0.001) showed less anxiety compared to WT mice, reflected by a higher percentage of time spent in the open arms of the elevated plus maze. Caffeine treatment did not impact this behavioural phenotype (Supplementary Fig. 3A), and no differences in the overall number of open arm entries were noted among all experimental groups (Supplementary Fig. 3B).

### Caffeine rescues both recognition and spatial memory deficits in Tg4-42 and 5xFAD mice

The open field test represents the habituation phase for the novel object recognition (NOR) and the novel object location (NOL) tasks (Supplementary Fig. 4A), which were used to analyse object recognition memory and location preference in untreated and caffeine-treated Tg4-42 and 5xFAD mice. While no obvious differences with regard to the time spent in the centre were observed among all experimental groups (Supplementary Fig. 4B), 5xFAD mice travelled a shorter distance in this task when compared to both WT and Tg4-42 mice (*p* < 0.05) (Supplementary Fig. 4C).

**Fig. 2:**
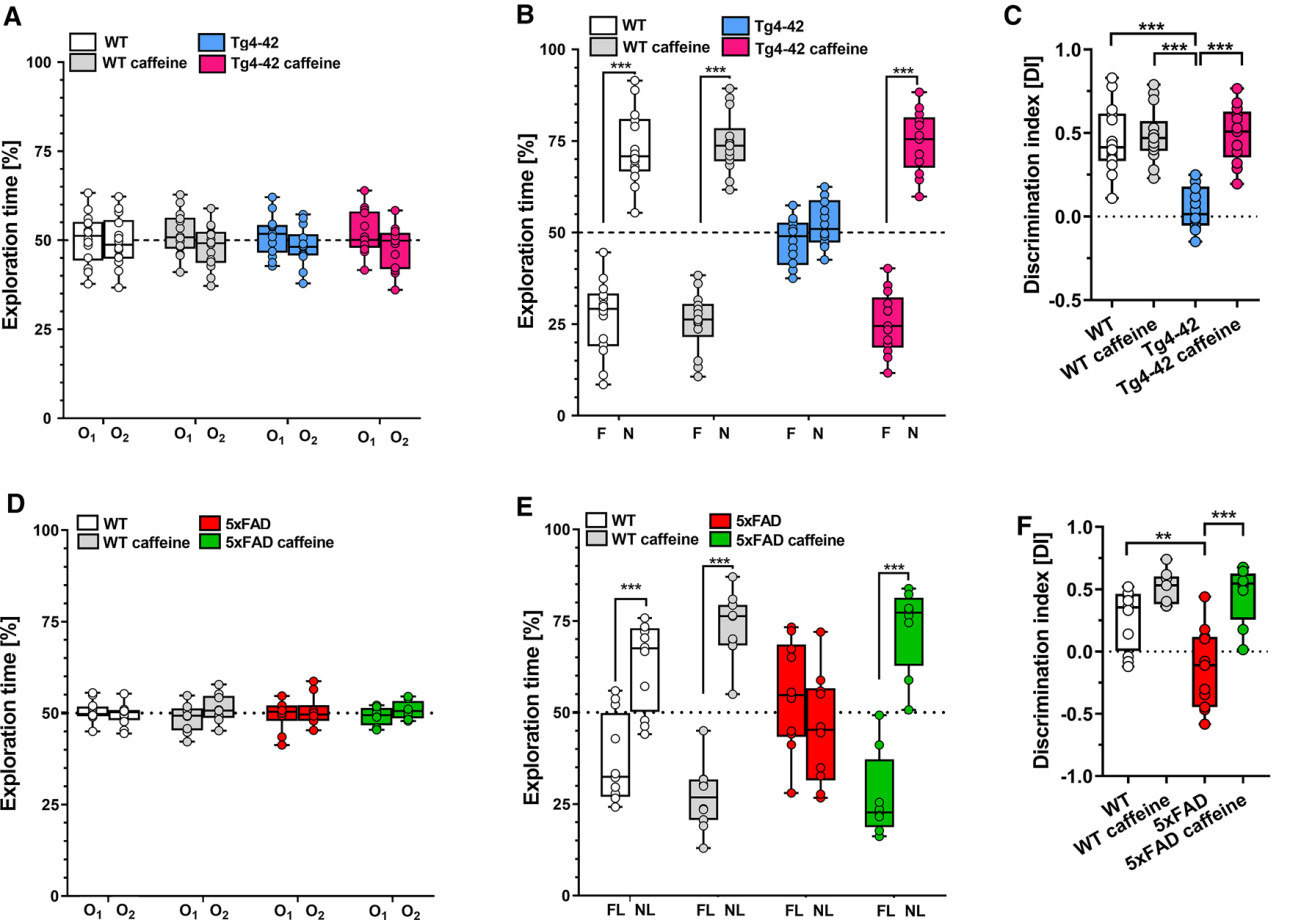
Long-term oral caffeine treatment ameliorated memory deficits in 6-month-old Tg4-42 and 5xFAD mice. During the training phase on the first day (**A**, **D**), all experimental groups spent an equal amount of time exploring each of the similar objects (O_1_, O_2_). During the testing phase 24 h later, untreated Tg4-42 and 5xFAD mice did not show a preference for any of the two objects. In contrast, caffeine-treated Tg4-42 (**B**) and 5xFAD (**E**) mice spent significantly more time with the novel object (N) or with the object placed in the new location (NL). Calculation of the discrimination index (DI), showed significantly higher scores for both caffeine-treated Tg4-42 (**C**) and 5xFAD mice (**F**) compared to the untreated groups (*n* = 14 per group). Two-way ANOVA (**A**, **B**, **D**, **E**) or One-way ANOVA (**C**, **F**) followed by Tukey’s multiple comparisons tests. **p < 0.01, ***p < 0.001. Data are presented as means ± SD

On the exploration day, the animals in all groups explored two identical objects equally (Fig. 2A). When tested for recognition memory 24 h later, untreated Tg4-42 mice did not show a preference for any of the two objects while caffeine-treated Tg4-42 mice engaged significantly longer with the novel object, resulting in a performance similar to the level of WT animals (Fig. 2B, *p* < 0.001).

Spatial memory deficits in hippocampus-related tasks such as novel object location have been described in 5xFAD mice [41]. In the NOL test, spatial long-term memory was evaluated. On the first day, mice were trained in the presence of two indistinguishable objects (O_1_ and O_2_), and no difference in the exploration time could be measured between any of the groups (Fig. 2D). When tested for spatial memory 24 h later, caffeine-treated 5xFAD mice exhibited a significantly increased preference for the displaced object (novel location, NL) compared to the untreated control group (Fig. 2E, *p* < 0.001). A calculation of the discrimination index (DI) showed significantly higher values for both the caffeine-treated Tg4-42 and 5xFAD mice compared to the respective untreated control groups (Fig. 2C, F, both *p* < 0.001, respectively).

### Caffeine rescues spatial reference memory in Tg-4-42 mice

**Fig. 3:**
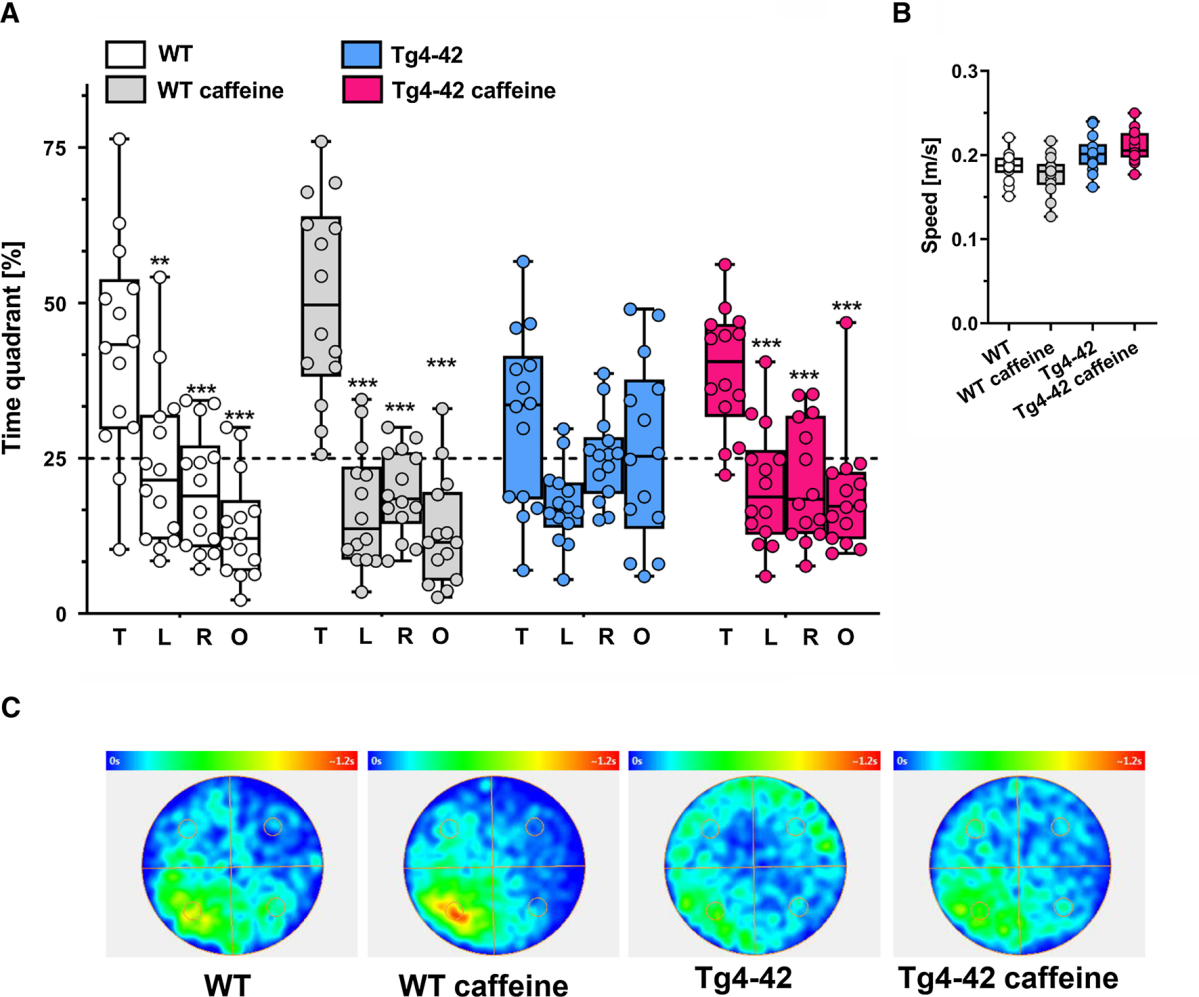
Spatial reference memory of Tg4-42 mice was improved by caffeine in the Morris water maze task. **A** While untreated Tg4-42 mice did not show a preference for any of the quadrants in the probe trial, caffeine-treated Tg4-42 and WT mice presented with intact spatial reference memory, spending significantly more time in the target quadrant (T) compared with all other quadrants (L, left; R, right; O, opposite). **B** No differences in the swimming speed were detected between the experimental groups during the probe trial. **C** Occupancy plots are shown illustrating the averaged swimming traces of untreated and caffeine-treated animals during the probe trial (*n* = 14 per group). **A** Two-way ANOVA with Tukey’s multiple comparison tests; **p < 0.01; ***p < 0.001. Data are presented as means ± SD

At 6 months of age, Tg4-42 mice displayed a spatial reference memory deficit in the Morris water maze (MWM) task, as demonstrated previously [27]. Over 3 days of cued training, all groups showed decreased escape latencies, although Tg4-42 mice performed significantly different from WT mice (Supplementary Fig. 5A). This was also observed in the 5 days of acquisition training with WT mice showing shorter escape latencies compared to Tg4-42 mice. Interestingly, caffeine-treated WT mice showed an even further reduced escape latency compared to untreated WT mice in the acquisition phase (Supplementary Fig. 5C). While initial swimming speed was somewhat slower for caffeine-treated and untreated Tg4-42 mice compared to their respective WT control groups (Supplementary Fig. 5B; *p* < 0.05), all groups showed comparable swimming speeds during the acquisition training period (Supplementary Fig. 5D). In the probe trial, untreated Tg4-42 mice showed no clear preference for the target quadrant, while caffeine-treated Tg4-42 mice showed preserved spatial reference memory, performing at levels similar to WT mice (Fig. 3A; *p* < 0.001). Importantly, no differences in swimming speed were observed among all groups during the probe trial (Fig. 3B). Representative occupancy plots confirmed a more focused search strategy in caffeine-treated Tg4-42 mice towards the initial platform position, while untreated mice displayed a more random pattern (Fig. 3C). Untreated Tg4-42 mice show a significantly reduced number of goal quadrant entries compared to WT (*t* test, mean difference − 1.86 ± 0.7 [95% CI − 3.297, − 0.417, *p* < 0.05), confirming their reduced performance in this task, while caffeine-treated Tg4-42 mice showed significantly more target quadrant entries (Supplementary Fig. 5E; mean difference 1.79 [95% CI 0.5, 2.93], *p* < 0.05) and reduced latencies of the initial target quadrant entry in the probe trial (Supplementary Fig. 5F; mean difference -5.46 [95% CI − 9.61, − 2.06], *p* < 0.05) in comparison to the untreated Tg4-42 group.

### Caffeine decreases hippocampal neuron loss and ameliorates impaired neurogenesis in Tg4-42 mice

**Fig. 4:**
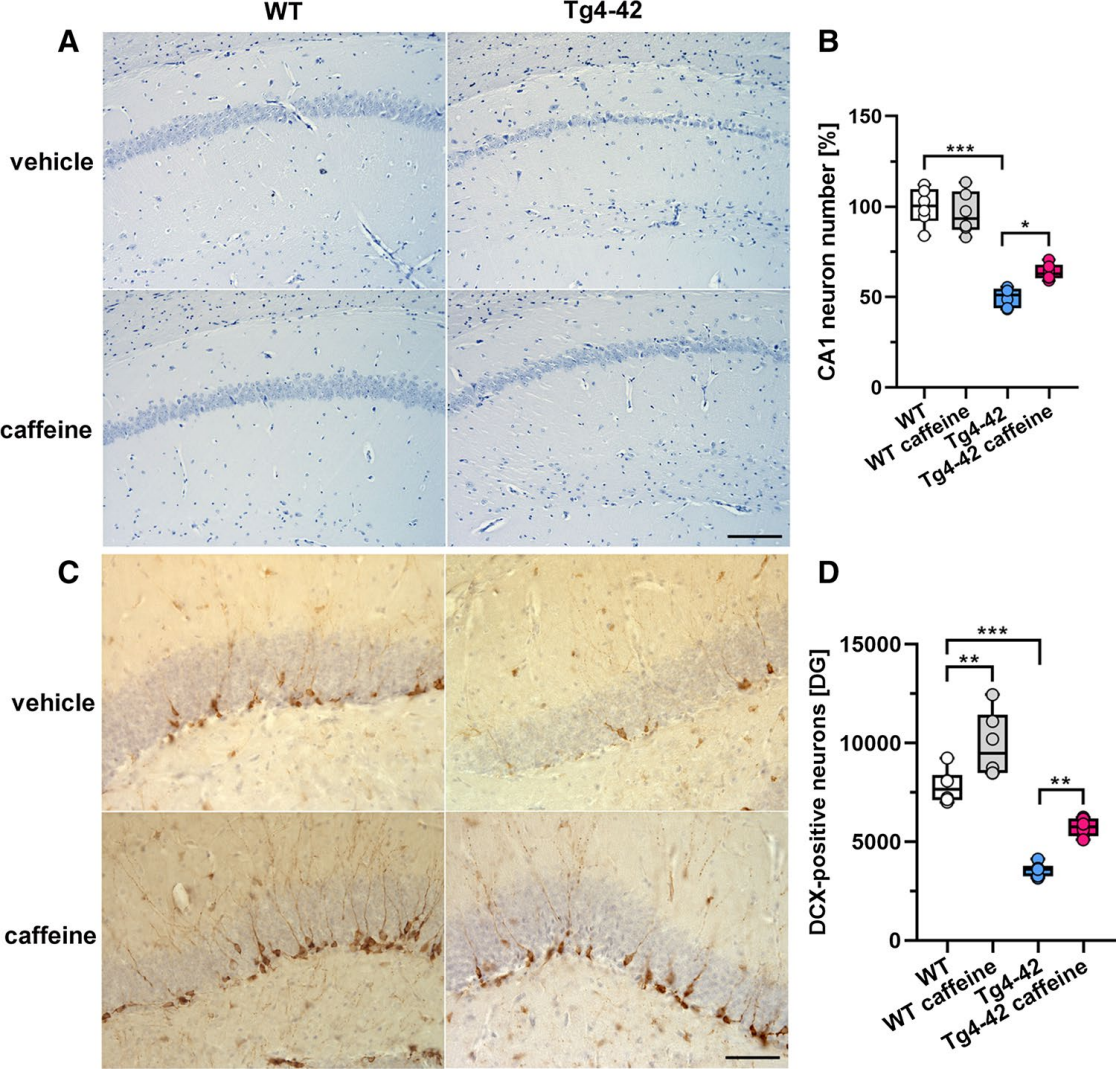
Chronic caffeine treatment ameliorated CA1 neuron loss and rescued neurogenesis in Tg4-42 mice. Quantification of haematoxylin-stained neurons revealed significantly reduced CA1 neuron counts in untreated Tg4-42 compared to WT mice, which was substantially ameliorated by caffeine treatment (**A**, **B**). In addition, significantly increased numbers of DCX-positive cells in the dentate gyrus were detected in both WT and Tg4-42 mice after long-term caffeine consumption (**C**, **D**) (*n* = 6 per group). One-way ANOVA with Tukey’s multiple comparison tests; *p < 0.05, **p < 0.01, ***p < 0.001. Data are presented as means ± SD. Scale bars: A = 200 µm; C = 50 µm

To assess whether chronic oral caffeine consumption might impact CA1 neuron loss in Tg4-42 mice, the number of haematoxylin-stained neuronal nuclei was quantified in a defined area of the hippocampal CA1 region in 6-month-old untreated and caffeine-treated WT and Tg4-42 mice. In good agreement with a previous study [27], 6-month-old Tg4-42 mice showed ~ 50% neuron loss compared to aged-matched WT animals (Fig. 4A, B; mean difference − 50.15 [95% CI − 36.36, − 63.94], *p* < 0.001). Compared to the untreated WT control group, caffeine-treated Tg4-42 mice displayed an ameliorated neuron loss of ~ 36% (mean difference 36.22 [95% CI 22.43, 50.00], *p* < 0.001). Importantly, a direct comparison between treated and untreated Tg4-42 mice showed a ~ 28% higher CA1 pyramidal neuron number in caffeine-treated Tg4-42 mice (Fig. 4B; mean difference 13.93 [95% CI 0.147, 27.27], *p* < 0.05). Stereological analysis of Doublecortin (DCX)-positive cells in the dentate gyrus of the hippocampus revealed a strongly reduced neuron number in 6-month-old Tg4-42 compared to WT mice (Fig. 6C, D; mean difference − 4227 [95% CI − 5786, − 2667], *p* < 0.001). Long-term oral caffeine treatment increased the number of DCX-positive cells in both WT (mean difference 2113 [95% CI 554, 3673], *p* < 0.01) and Tg4-42 mice (mean difference 2152 [95% CI 592, 3711], *p* < 0.01) in comparison to their untreated control groups (Fig. 4D).

### Caffeine does not influence amyloid deposition, APP processing or astrocytosis in the brains of 5xFAD mice

**Fig. 5:**
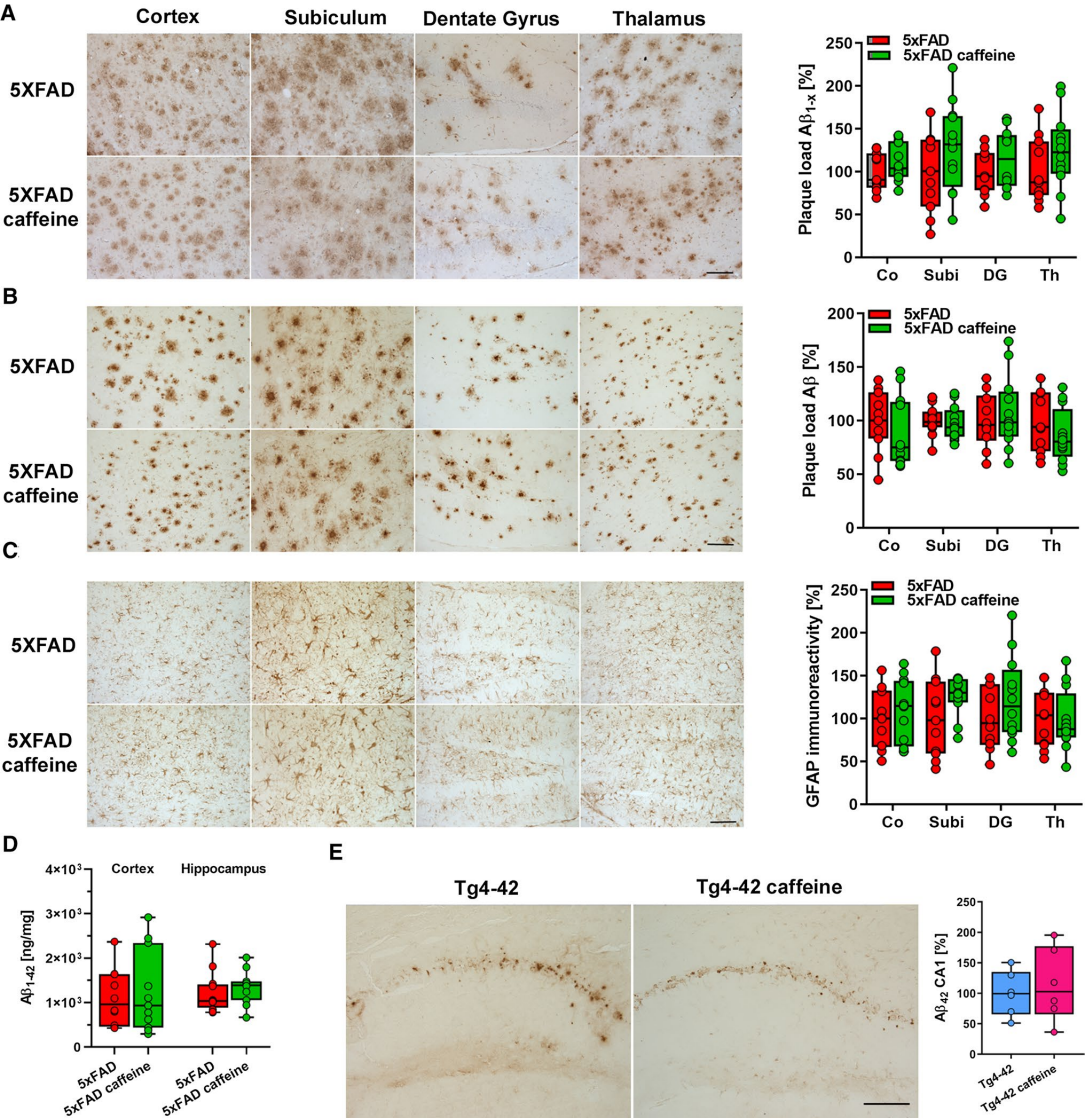
Caffeine treatment did not affect amyloid pathology and astrogliosis. Quantification of the Aβ plaque load in the cortex (Co), subiculum (Subi), dentate gyrus (DG) and thalamus (Th) of 6-month-old 5xFAD mice using a pan-Aβ antibody **A** and a N-terminal specific antibody **B** showed that caffeine treatment did not alter Aβ plaque deposition in any of the brain regions. Staining with the astrocytic marker GFAP revealed comparable immunoreactivity in all four brain regions in untreated and caffeine-treated 5xFAD mice (*n* = 10–12 per group) (**C**). Conforming to the amyloid plaque load analysis, no differences in SDS-soluble Aβ_1–42_ levels were detected in the cortex and hippocampus using ELISA (*n* = 11–12 per group) (**D**). Quantification of Aβ immunoreactivity in the CA1 region of 6-month-old Tg4-42 mice showed no changes upon caffeine treatment (*n* = 6 per group). Two-way ANOVA (**A**-**C**) or unpaired *t* tests (**D**, **E**). Data are given as means ± SD. Scale bar = 100 μm

5xFAD mice present with extracellular amyloid pathology starting at ~ 2 months of age in the hippocampus, cortex, and thalamus [30, 31]. To evaluate if chronic caffeine treatment would have an impact on amyloid deposition, the plaque pathology was quantified in the cortex, subiculum, dentate gyrus and thalamus of untreated and caffeine-treated 5xFAD mice. Sagittal brain sections of 6-month-old mice were stained with Aβ antibodies detecting total Aβ (24311, pan-Aβ) or full-length Aβ species starting with the aspartic acid at position 1 (82E1, Aβ_1-x_). No differences in the extent of the extracellular amyloid deposition were detected between caffeine-treated and untreated mice in all brain areas analysed (Fig. 5A, B).

To biochemically evaluate whether Aβ levels were altered in a treatment-dependent manner, Aβ_1–42_ levels were measured in the SDS-soluble protein fractions of brain cortices and hippocampi by ELISA. No differences were detected between the experimental groups (Fig. 5D). In the Tg4-42 mouse model, Aβ_4–42_ accumulation is predominantly detectable in the CA1 layer of the hippocampus. In agreement with the data obtained in 5xFAD mice, immunohistochemical quantification of Aβ levels revealed no differences between caffeine-treated and untreated mice (Fig. 5E).

**Fig. 6:**
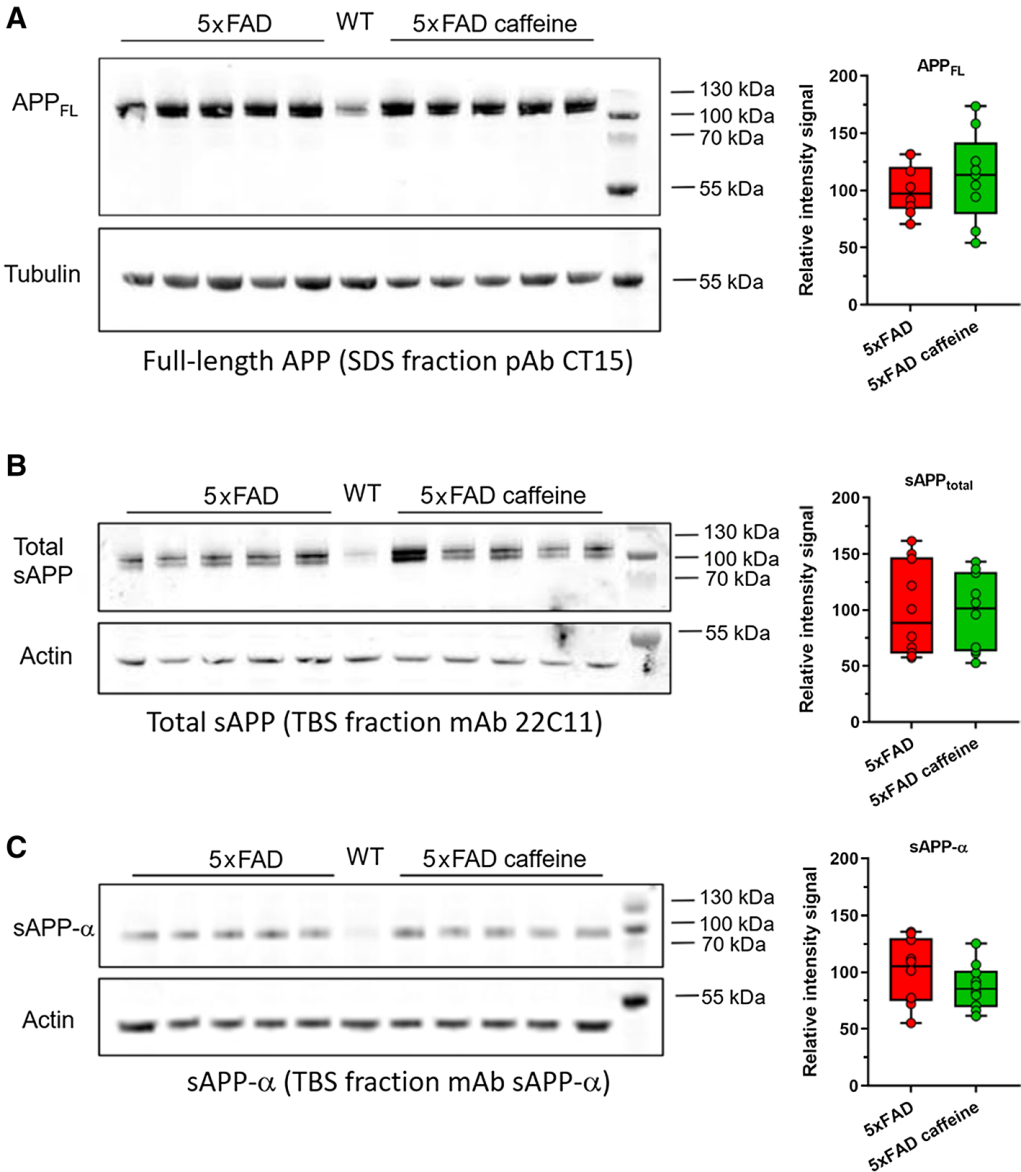
Analysis of APP processing in 6-month-old 5xFAD mice after chronic oral caffeine treatment. No changes were observed in the levels of full-length APP in hippocampal, SDS-soluble brain fractions of untreated and caffeine-treated 5XFAD mice **A**. Likewise, in TBS-soluble brain fractions, total levels of the soluble APP ectodomain (sAPP) **B** and levels of the soluble APP ectodomain generated by α-secretase cleavage of APP (sAPP-α) **C** were not affected by caffeine treatment. WT mice lack the human APP-transgene and express only endogenous levels of murine APP. Each Western blot was repeated three times and one representative blot is shown. For quantification, APP or sAPP signal intensities were measured, normalized to either tubulin or actin levels, and averaged for the three technical replicates. Means were calculated for each treatment group (*n* = 5 per group), and the values of the untreated animals were set to 100%. Unpaired t tests. All data are given as means ± SD

To determine whether chronic caffeine treatment modulates APP processing in vivo, soluble and cell-associated metabolites of APP were measured in the hippocampi of caffeine-treated and untreated 5xFAD mice by Western blotting. Full-length APP levels were measured in the SDS-soluble brain fractions (Fig. 6A), while total levels of the soluble APP ectodomain (sAPP) and levels of the soluble APP ectodomain generated by α-secretase cleavage of APP (sAPP-α) were determined in the TBS-soluble brain fractions (Fig. 6B, C, respectively). Quantification of full-length APP, sAPP, and sAPP-α levels did not show differences between the experimental groups, indicating that caffeine treatment did not affect APP processing.

To evaluate whether caffeine consumption impacts disease-associated astrogliosis as one aspect of neuroinflammatory pathology [58], reactive astrocytes with glial fibrillary acidic protein (GFAP) as a marker were quantified [59]. No differences in GFAP signal intensities were detected in the cortex, dentate gyrus and thalamus between untreated and caffeine-treated 5xFAD mice at 6 months of age (Fig. 5C), which is in good agreement with unchanged Aβ pathology.

### RNA sequencing of hippocampal neurons in Tg4-42 mice

**Fig. 7:**
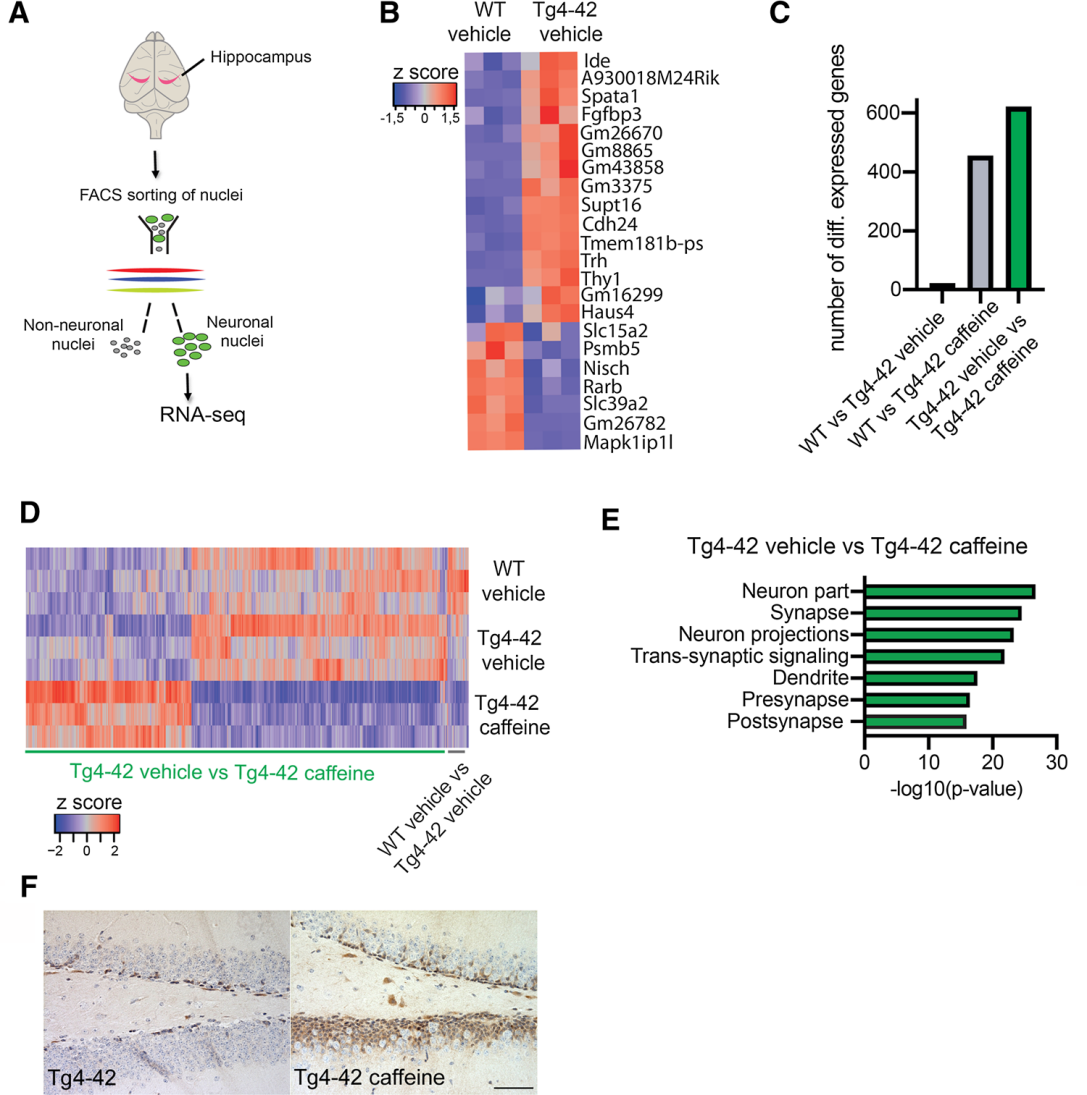
Gene expression analysis in caffeine-treated Tg4-42 mice. **A** Scheme showing the experimental design of the RNA sequencing analysis with hippocampal neuronal nuclei. **B** Heat map showing the 22 hippocampal genes that were differentially expressed between vehicle-treated WT and Tg4-42 mice (FDR < 0.05). **C** Bar chart showing the number of differentially expressed genes for the indicated comparisons. **D** Heat map showing the 622 genes differentially expressed between vehicle- and caffeine-treated Tg4-42 mice. In addition, the 22 genes differentially expressed between vehicle-treated WT and Tg4-42 mice are displayed across all 3 analyzed groups. **E** GO-term analysis of the 622 genes differentially expressed between vehicle- and caffeine-treated Tg4-42 mice. **F** Immunohistochemical analysis of the cell–cell contact protein Desmoplakin (Dsp). The Dsp gene showed the strongest upregulation in caffeine-treated compared to vehicle-treated Tg4-42 mice. Abundant immunoreactivity was observed in the dentate gyrus granule cell layer of caffeine- but not vehicle-treated Tg4-42 mice. Scale bar: 50 µm

To further investigate whether transcriptional changes might underlie the partial rescue of cognitive functions in Tg4-42 mice treated with caffeine, we isolated neuronal nuclei from the hippocampi of Tg4-42 mice treated with caffeine or vehicle as well as from vehicle-treated WT mice, and performed RNA sequencing (Fig. 7A). When comparing vehicle-treated WT to corresponding Tg4-42 mice, we observed only minor changes in gene expression with only 22 genes that were differentially expressed (Fig. 7B; Supplemental Table 1). Of interest, the *Ide* gene coding for insulin-degrading enzyme was up-regulated in Tg4-42 mice, which has been linked to the clearance of Aβ peptides [60]. The up-regulation of the *Trh* and *Thy1* genes was expected and can be explained by the organization of the promoter construct used to drive Aβ4–42 expression [33]. These data suggest that neuronally produced intracellular Aβ_4–42_ peptides only have a minor effect on transcriptional homeostasis. In contrast, caffeine administration altered the expression of many genes (Fig. 7C, D; Supplemental Tables 2,3). GO analysis of the 622 genes differentially expressed between vehicle- and caffeine-treated Tg4-42 mice revealed that they almost exclusively represent key mechanisms linked to synaptic function and processes, as well as to neural progenitor proliferation (Fig. 7E; Supplemental Table 4). These data suggest that caffeine might induce a therapeutic gene expression response that contributes to the maintenance of cognitive functions in Tg4-42 mice. Immunohistochemical staining of the cell–cell contact protein Desmoplakin (Dsp), encoded by the most strongly up-regulated gene in caffeine-treated animals, revealed abundant immunoreactivity mainly in the dentate gyrus, providing confirmation for the transcriptome analysis on the protein level (Fig. 7F).

## Discussion

In this study, caffeine was administered in a dosage of 0.3 mg/ml in the drinking water for a duration of 4 months. With an average consumption of ~ 5 ml of caffeinated water per day, this results in a daily intake of 1.5 mg caffeine per mouse. Due to the higher metabolic rate in mice, this corresponds to approx. 500 mg in humans (an equivalent of ~ 5 cups per day) [20]. Consistent with previous studies reporting beneficial effects of caffeine on memory in rodent preclinical mouse models of dementia [14–16, 20, 34], we observed a significantly improved performance in spatial reference memory tasks such as the MWM in caffeine-treated Tg4-42 mice. Analysis of object recognition memory also revealed a significant improvement in either the NOR (Tg4-42) or the NOL task (5XFAD) upon long-term caffeine treatment, resulting in a complete rescue of the deficits observed in both untreated transgenic lines.

A recent PIB-PET imaging study in non-demented older adults reported that a lifetime coffee intake of 2 or more cups per day was significantly associated with lower brain Aβ positivity, suggesting a direct relationship between coffee consumption and APP metabolism [10] and levels of the caffeine metabolite theobromine showed a significant correlation with CSF Aβ42 levels in AD and MCI patients [9]. However, in contrast to several previous studies conducted in other mouse models, we did not detect a significant reduction in brain Aβ levels in either 5xFAD or Tg4-42 mice upon caffeine treatment with the chosen treatment paradigm. Previously, significantly reduced levels of Aβ_1–40_ in the soluble and Aβ_1–42_ in the insoluble brain fraction were reported in the hippocampus of 9-month-old APPsw mice treated for 5.5 months [20], as well as in 21-month-old APPsw mice treated for 4–5 weeks [19, 21]. Mechanistically, a recent *in vitro* study with APP695-transfected SH-SY5Y neuroblastoma cells reported that caffeine treatment decreased Aβ levels by shifting APP processing towards the non-amyloidogenic pathway with increased α- and decreased β-secretase activity [61].

In our study, neither the extracellular amyloid plaque load in different brain regions nor Aβ_1–42_ levels measured in cortical and hippocampal tissue lysates showed any evidence of a reduction upon caffeine treatment. We were also unable to detect any alterations in APP processing upon caffeine treatment in the brains of 5xFAD mice, with unchanged levels of full-length APP, total sAPP and sAPP-α. This is in contrast with previous studies [19–21] and might be explained by the differences in the experimental models, with earlier, more rapid and robust extracellular Aβ deposition in the 5xFAD model used in the present study [30, 31, 62].

Beyond a possible direct impact of caffeine on APP metabolism, there is evidence from transgenic and non-transgenic AD mouse models that antagonism of adenosine receptors to control synaptic function and plasticity plays a major role in the beneficial effects of caffeine in AD. While this relationship has been extensively studied [15, 16, 63–66], potential effects of caffeine on neurogenesis are less clear.

Intriguingly, long-term oral caffeine treatment of Tg4-42 mice resulted in a substantially reduced neuron loss in the CA1 region of the hippocampus. In both WT and Tg4-42 mice treated with caffeine, we further observed significantly increased numbers of DCX-positive neural progenitor cells in the DG in comparison to the vehicle-treated littermates. However, the data in the literature are somewhat contradictory. In in vitro experiments, acute caffeine exposure compromised the proliferation of human hippocampal progenitor cells [67]. In addition, 4-week caffeine consumption reduced the number of hippocampal neural precursors and impaired learning and memory in rats [68], while it improved recognition memory without altering the number of newborn neurons in the DG in a bred-based model of depression [25]. A 7-day administration of moderate to high doses of caffeine depressed the proliferation of adult hippocampal precursor cells in adult mice [69]. However, the difference to our observation of increased neurogenesis rates in both caffeine-treated WT and Tg4-42 mice might be attributed to the chronic treatment paradigm applied in our study. More in line with our data, intragastric administration of caffeine in mice has been demonstrated to revert a block in adult hippocampal neurogenesis caused by chronic water immersion restraint stress [70], and caffeine treatment during sleep deprivation significantly increased early proliferative and post-mitotic stages of DCX-positive cells in the murine hippocampus [71]. Importantly, the neurogenic effects on DCX-positive neural progenitor cells in caffeine-treated Tg4-42 mice were also reflected in the results of the hippocampal neuron transcriptome analysis. In this analysis, only a limited number of genes appeared to be differentially expressed between WT and untreated Tg4-42 mice. In APP-overexpressing transgenic mice that model amyloid deposition, much more substantial changes in gene expression have been observed [72]. This might be explained by the fact that the Aβ peptide is specifically overexpressed within neurons in the Tg4-42 model, thus representing mainly the effects of intracellular Aβ accumulation. Moreover, we specifically analyzed the neuronal transcriptome. A variety of genes upregulated upon caffeine treatment were associated with neural progenitor proliferation, including Desmoplakin (Dsp), prospero homeobox 1 (Prox1) and Bcl11B/Ctip2. Ctip2 and Prox1expression have previously been reported to be upregulated by caffeine treatment in sheep and rats [73]. Dsp, a cell–cell contact gene, is a direct transcriptional target of Bcl11B/Ctip2 [74] and was the gene most strongly upregulated upon caffeine treatment in our data set. Furthermore, Dsp was strongly down-regulated in Norbin-deficient mice, which present with significantly reduced hippocampal neurogenesis [75]. The observed effects on neurogenesis also fit well with the observation that beneficial effects of caffeine treatment are associated with increased plasma levels of granulocyte-colony stimulating factor (GCSF) [76], as this factor was reported to function as a neuronal ligand that drives neurogenesis [77]. The improvements in learning and memory-related parameters such as the discrimination index, together with increased neurogenesis rates supported earlier findings of a quantitative relationship between the hippocampal neurogenesis and the extent of memory dysfunction [78]. However, our study also has several limitations. In our RNA sequencing analysis, the entire hippocampus has been analysed without considering hippocampal dorso-ventral functional heterogeneity. While the dorsal region is mainly involved in learning and memory processes related to spatial navigation and locomotion, the ventral part is primarily associated with emotion or motivation [79–81]. Another point to consider is a potential influence of the circadian cycle. Though we tried to ensure that all animals were sacrificed in a rather narrow and constant time frame, we cannot rule out that that gene and protein expression display circadian oscillations among the lines due to disease conditions, as it has been shown e.g. in experimental epilepsy [82, 83]. Finally, we specifically analyzed neuronal nuclei and thus may not be able to detect other relevant processes such as inflammatory responses in microglia.

In conclusion, prolonged oral caffeine treatment ameliorated neuron loss and learning and memory deficits, and promoted cellular and molecular markers of neurogenesis in the absence of detectable effects on the Aβ pathology in two transgenic AD mouse models.

## Supplementary Information

Below is the link to the electronic supplementary material.

Supplementary file1 (PDF 729 KB)

Supplementary file2 (XLSX 137 KB)

## Data Availability

All primary data and material in the manuscript are available upon reasonable request. Gene expression data have been submitted to the Gene Expression Omnibus (GEO) database under accession number GSE183323.

## References

[1] Cunha RA (2016). How does adenosine control neuronal dysfunction and neurodegeneration?. J Neurochem.

[2] Ding M, Bhupathiraju SN, Chen M, van Dam RM, Hu FB (2014). Caffeinated and decaffeinated coffee consumption and risk of type 2 diabetes: a systematic review and a dose-response meta-analysis. Diabetes Care.

[3] Ding M, Satija A, Bhupathiraju SN, Hu Y, Sun Q, Han J, Lopez-Garcia E, Willett W, van Dam RM, Hu FB (2015). Association of coffee consumption with total and cause-specific mortality in 3 large prospective cohorts. Circulation.

[4] Madeira MH, Boia R, Ambrósio AF, Santiago AR (2017). Having a coffee break: the impact of caffeine consumption on microglia-mediated inflammation in neurodegenerative diseases. Mediat Inflamm.

[5] Ritchie K, Carrière I, de Mendonça A, Portet F, Dartigues JF, Rouaud O, Barberger-Gateau P, Ancelin ML (2007). The neuroprotective effects of caffeine: a prospective population study (the Three City Study). Neurology.

[6] van Gelder BM, Buijsse B, Tijhuis M, Kalmijn S, Giampaoli S, Nissinen A, Kromhout D (2007). Coffee consumption is inversely associated with cognitive decline in elderly European men: the FINE Study. Eur J Clin Nutr.

[7] Zhou X, Zhang L (2021). The neuroprotective effects of moderate and regular caffeine consumption in Alzheimer’s disease. Oxid Med Cell Longev.

[8] Cao C, Loewenstein DA, Lin X, Zhang C, Wang L, Duara R, Wu Y, Giannini A, Bai G, Cai J, Greig M, Schofield E, Ashok R, Small B, Potter H, Arendash GW (2012). High blood caffeine levels in MCI linked to lack of progression to dementia. J Alzheimers Dis.

[9] Travassos M, Santana I, Baldeiras I, Tsolaki M, Gkatzima O, Sermin G, Yener GG, Simonsen A, Hasselbalch SG, Kapaki E, Mara B, Cunha RA, Agostinho P, Blennow K, Zetterberg H, Mendes VM, Manadas B, de Mendon A (2015). Does caffeine consumption modify cerebrospinal fluid amyloid-β levels in patients with Alzheimer’s disease?. J Alzheimers Dis.

[10] Kim JW, Byun MS, Yi D, Lee JH, Jeon SY, Jung G, Lee HN, Sohn BK, Lee J-Y, Kim YK, Shin SA, Sohn C-H, Lee DY (2019). Coffee intake and decreased amyloid pathology in human brain. Transl Psychiatry.

[11] Eskelinen MH, Ngandu T, Tuomilehto J, Soininen H, Kivipelto M (2009). Midlife coffee and tea drinking and the risk of late-life dementia: a population-based CAIDE study. J Alzheimers Dis.

[12] Maia L, De Mendonça A (2002). Does caffeine intake protect from Alzheimer's disease?. Eur J Neurol.

[13] Londzin P, Zamora M, Kąkol B, Taborek A, Folwarczna J (2021). Potential of caffeine in Alzheimer's disease-a review of experimental studies. Nutrients.

[14] Han K, Jia N, Li J, Yang L, Min LQ (2013). Chronic caffeine treatment reverses memory impairment and the expression of brain BNDF and TrkB in the PS1/APP double transgenic mouse model of Alzheimer's disease. Mol Med Rep.

[15] Dall'Igna OP, Fett P, Gomes MW, Souza DO, Cunha RA, Lara DR (2007). Caffeine and adenosine A(2a) receptor antagonists prevent beta-amyloid (25–35)-induced cognitive deficits in mice. Exp Neurol.

[16] Espinosa J, Rocha A, Nunes F, Costa MS, Schein V, Kazlauckas V, Kalinine E, Souza DO, Cunha RA, Porciúncula LO (2013). Caffeine consumption prevents memory impairment, neuronal damage, and adenosine a 2A receptors upregulation in the hippocampus of a rat model of sporadic dementia. J Alzheimers Dis.

[17] Lopes JP, Pliássova A, Cunha RA (2019). The physiological effects of caffeine on synaptic transmission and plasticity in the mouse hippocampus selectively depend on adenosine A1 and A2A receptors. Biochem Pharmacol.

[18] Kaster MP, Machado NJ, Silva HB, Nunes A, Ardais AP, Santana M, Baqi Y, Müller CE, Rodrigues ALS, Porciúncula LO, Chen JF, Tomé ÂR, Agostinho P, Canas PM, Cunha RA (2015). Caffeine acts through neuronal adenosine A2A receptors to prevent mood and memory dysfunction triggered by chronic stress. Proc Natl Acad Sci USA.

[19] Arendash GW, Mori T, Cao C, Mamcarz M, Runfeldt M, Dickson A, Rezai-Zadeh K, Tan J, Citron BA, Lin X, Echeverria V, Potter H (2009). Caffeine reverses cognitive impairment and decreases brain amyloid-β levels in aged Alzheimer's disease mice. J Alzheimers Dis.

[20] Arendash GW, Schleif W, Rezai-Zadeh K, Jackson EK, Zacharia LC, Cracchiolo JR, Shippy D, Tan J (2006). Caffeine protects Alzheimer’s mice against cognitive impairment and reduces brain β-amyloid production. Neuroscience.

[21] Cao C, Cirrito JR, Lin X, Wang L, Verges DK, Dickson A, Mamcarz M, Zhang C, Mori T, Arendash GW, Holtzman DM, Potter H (2009). Caffeine suppresses amyloid-β levels in plasma and brain of Alzheimer's disease transgenic mice. J Alzheimers Dis.

[22] Pandolfo P, Machado NJ, Köfalvi A, Takahashi RN, Cunha RA (2013). Caffeine regulates frontocorticostriatal dopamine transporter density and improves attention and cognitive deficits in an animal model of attention deficit hyperactivity disorder. Eur Neuropsychopharmacol.

[23] Duarte JMN, Agostinho PM, Carvalho RA, Cunha RA (2012). Caffeine consumption prevents diabetes-induced memory impairment and synaptotoxicity in the hippocampus of NONcZNO10/LTJ mice. PLoS ONE.

[24] Cognato GP, Agostinho PM, Hockemeyer J, Müller CE, Souza DO, Cunha RA (2010). Caffeine and an adenosine A2A receptor antagonist prevent memory impairment and synaptotoxicity in adult rats triggered by a convulsive episode in early life. J Neurochem.

[25] Machado NJ, Simões AP, Silva HB, Ardais AP, Kaster MP, Garção P, Rodrigues DI, Pochmann D, Santos AI, Araújo IM, Porciúncula LO, Tomé ÂR, Köfalvi A, Vaugeois J-M, Agostinho P, El Yacoubi M, Cunha RA, Gomes CA (2017). Caffeine reverts memory but not mood impairment in a depression-prone mouse strain with up-regulated adenosine A2A receptor in hippocampal glutamate synapses. Mol Neurobiol.

[26] Portelius E, Andreasson U, Ringman J, Buerger K, Daborg J, Buchhave P, Hansson O, Harmsen A, Gustavsson M, Hanse E, Galasko D, Hampel H, Blennow K, Zetterberg H (2010). Distinct cerebrospinal fluid amyloid beta peptide signatures in sporadic and PSEN1 A431E-associated familial Alzheimer's disease. Mol Neurodegen.

[27] Antonios G, Borgers H, Richard BC, Brauß A, Meißner J, Weggen S, Pena V, Pillot T, Davies SL, Bakrania P, Matthews D, Brownlees J, Bouter Y, Bayer TA (2015). Alzheimer therapy with an antibody against N-terminal Abeta 4-X and pyroglutamate Abeta 3-X. Sci Rep.

[28] Stazi M, Wirths O (2021). Chronic memantine treatment ameliorates behavioral deficits, neuron loss, and impaired neurogenesis in a model of Alzheimer’s disease. Mol Neurobiol.

[29] Gerberding A-L, Zampar S, Stazi M, Liebetanz D, Wirths O (2019). Physical activity ameliorates impaired hippocampal neurogenesis in the Tg4-42 mouse model of Alzheimer's disease. ASN Neuro.

[30] Oakley H, Cole SL, Logan S, Maus E, Shao P, Craft J, Guillozet-Bongaarts A, Ohno M, Disterhoft J, Van Eldik L, Berry R, Vassar R (2006). Intraneuronal beta-amyloid aggregates, neurodegeneration, and neuron loss in transgenic mice with five familial Alzheimer's disease mutations: potential factors in amyloid plaque formation. J Neurosci.

[31] Jawhar S, Trawicka A, Jenneckens C, Bayer TA, Wirths O (2012). Motor deficits, neuron loss, and reduced anxiety coinciding with axonal degeneration and intraneuronal Abeta aggregation in the 5XFAD mouse model of Alzheimer's disease. Neurobiol Aging.

[32] Hüttenrauch M, Ogorek I, Klafki H, Otto M, Stadelmann C, Weggen S, Wiltfang J, Wirths O (2018). Glycoprotein NMB: a novel Alzheimer’s disease associated marker expressed in a subset of activated microglia. Acta Neuropathol Commun.

[33] Bouter Y, Dietrich K, Wittnam JL, Rezaei-Ghaleh N, Pillot T, Papot-Couturier S, Lefebvre T, Sprenger F, Wirths O, Zweckstetter M, Bayer TA (2013). N-truncated amyloid β (Aβ) 4–42 forms stable aggregates and induces acute and long-lasting behavioral deficits. Acta Neuropathol.

[34] Laurent C, Eddarkaoui S, Derisbourg M, Leboucher A, Demeyer D, Carrier S, Schneider M, Hamdane M, Müller CE, Buée L, Blum D (2014). Beneficial effects of caffeine in a transgenic model of Alzheimer's disease-like tau pathology. Neurobiol Aging.

[35] Shiotsuki H, Yoshimi K, Shimo Y, Funayama M, Takamatsu Y, Ikeda K, Takahashi R, Kitazawa S, Hattori N (2010). A rotarod test for evaluation of motor skill learning. J Neurosci Methods.

[36] Karl T, Pabst R, von Horsten S (2003). Behavioral phenotyping of mice in pharmacological and toxicological research. Exp Toxicol Pathol.

[37] Antunes M, Biala G (2012). The novel object recognition memory: neurobiology, test procedure, and its modifications. Cogn Process.

[38] Hüttenrauch M, Walter S, Kaufmann M, Weggen S, Wirths O (2017). Limited effects of prolonged environmental enrichment on the pathology of 5XFAD mice. Mol Neurobiol.

[39] Park J-C, Ma J, Jeon WK, Han J-S (2016). Fructus mume extracts alleviate cognitive impairments in 5XFAD transgenic mice. BMC Complement Altern Med.

[40] Ardestani PM, Evans AK, Yi B, Nguyen T, Coutellier L, Shamloo M (2017). Modulation of neuroinflammation and pathology in the 5XFAD mouse model of Alzheimer's disease using a biased and selective beta-1 adrenergic receptor partial agonist. Neuropharmacology.

[41] de Pins B, Cifuentes-Díaz C, Farah AT, López-Molina L, Montalban E, Sancho-Balsells A, López A, Ginés S, Delgado-García JM, Alberch J, Gruart A, Girault J-A, Giralt A (2019). Conditional BDNF delivery from astrocytes rescues memory deficits, spine density, and synaptic properties in the 5xFAD mouse model of Alzheimer disease. J Neurosci.

[42] Denninger JK, Smith BM, Kirby ED (2018). Novel object recognition and object location behavioral testing in mice on a budget. JoVE.

[43] Leger M, Quiedeville A, Bouet V, Haelewyn B, Boulouard M, Schumann-Bard P, Freret T (2013). Object recognition test in mice. Nat Protoc.

[44] Morris R (1984). Developments of a water-maze procedure for studying spatial learning in the rat. J Neurosci Methods.

[45] Paxinos G, Franklin KBJ (2001). The mouse brain in stereotaxic coordinates.

[46] Zampar S, Wirths O (2021). Characterization of a mouse model of Alzheimer’s disease expressing Aβ4-42 and human mutant tau. Int J Mol Sci.

[47] Couillard-Despres S, Winner B, Schaubeck S, Aigner R, Vroemen M, Weidner N, Bogdahn U, Winkler J, Kuhn HG, Aigner L (2005). Doublecortin expression levels in adult brain reflect neurogenesis. Eur J Neurosci.

[48] Zampar S, Klafki HW, Sritharen K, Bayer TA, Wiltfang J, Rostagno A, Ghiso J, Miles LA, Wirths O (2020). N-terminal heterogeneity of parenchymal and vascular amyloid-β deposits in Alzheimer‘s disease. Neuropathol Appl Neurobiol.

[49] Saul A, Sprenger F, Bayer TA, Wirths O (2013). Accelerated tau pathology with synaptic and neuronal loss in a novel triple transgenic mouse model of Alzheimer's disease. Neurobiol Aging.

[50] Breyhan H, Wirths O, Duan K, Marcello A, Rettig J, Bayer TA (2009). APP/PS1KI bigenic mice develop early synaptic deficits and hippocampus atrophy. Acta Neuropathol.

[51] Walter S, Jumpertz T, Hüttenrauch M, Ogorek I, Gerber H, Storck SE, Zampar S, Dimitrov M, Lehmann S, Lepka K, Berndt C, Wiltfang J, Becker-Pauly C, Beher D, Pietrzik CU, Fraering PC, Wirths O, Weggen S (2019). The metalloprotease ADAMTS4 generates N-truncated Aβ4–x species and marks oligodendrocytes as a source of amyloidogenic peptides in Alzheimer’s disease. Acta Neuropathol.

[52] Jager S, Leuchtenberger S, Martin A, Czirr E, Wesselowski J, Dieckmann M, Waldron E, Korth C, Koo EH, Heneka M, Weggen S, Pietrzik CU (2009). alpha-secretase mediated conversion of the amyloid precursor protein derived membrane stub C99 to C83 limits Abeta generation. J Neurochem.

[53] Brockhaus M, Grunberg J, Rohrig S, Loetscher H, Wittenburg N, Baumeister R, Jacobsen H, Haass C (1998). Caspase-mediated cleavage is not required for the activity of presenilins in amyloidogenesis and NOTCH signaling. NeuroReport.

[54] Sakib MS, Sokpor G, Nguyen HP, Fischer A, Tuoc T (2021). Intranuclear immunostaining-based FACS protocol from embryonic cortical tissue. STAR Protocols.

[55] Ge SX, Jung D, Yao R (2020). ShinyGO: a graphical gene-set enrichment tool for animals and plants. Bioinformatics.

[56] Manouze H, Ghestem A, Poillerat V, Bennis M, Ba-M’hamed S, Benoliel JJ, Becker C, Bernard C (2019). Effects of single cage housing on stress, cognitive, and seizure parameters in the rat and mouse pilocarpine models of epilepsy. ENEURO.

[57] Ho J, Tumkaya T, Aryal S, Choi H, Claridge-Chang A (2019). Moving beyond P values: data analysis with estimation graphics. Nat Methods.

[58] Heneka MT, Carson MJ, Khoury JE, Landreth GE, Brosseron F, Feinstein DL, Jacobs AH, Wyss-Coray T, Vitorica J, Ransohoff RM, Herrup K, Frautschy SA, Finsen B, Brown GC, Verkhratsky A, Yamanaka K, Koistinaho J, Latz E, Halle A, Petzold GC, Town T, Morgan D, Shinohara ML, Perry VH, Holmes C, Bazan NG, Brooks DJ, Hunot S, Joseph B, Deigendesch N, Garaschuk O, Boddeke E, Dinarello CA, Breitner JC, Cole GM, Golenbock DT, Kummer MP (2015). Neuroinflammation in Alzheimer's disease. Lancet Neurol.

[59] Taipa R, Ferreira V, Brochado P, Robinson A, Reis I, Marques F, Mann DM, Melo-Pires M, Sousa N (2018). Inflammatory pathology markers (activated microglia and reactive astrocytes) in early and late onset Alzheimer disease: a post mortem study. Neuropathol Appl Neurobiol.

[60] Miners JS, Baig S, Palmer J, Palmer LE, Kehoe PG, Love S (2008). Abeta-degrading enzymes in Alzheimer's disease. Brain Pathol.

[61] Janitschke D, Nelke C, Lauer AA, Regner L, Winkler J, Thiel A, Grimm HS, Hartmann T, Grimm MOW (2019). Effect of caffeine and other methylxanthines on Aβ-homeostasis in SH-SY5Y cells. Biomolecules.

[62] Oblak AL, Lin PB, Kotredes KP, Pandey RS, Garceau D, Williams HM, Uyar A, O’Rourke R, O’Rourke S, Ingraham C, Bednarczyk D, Belanger M, Cope ZA, Little GJ, Williams S-PG, Ash C, Bleckert A, Ragan T, Logsdon BA, Mangravite LM, Sukoff Rizzo SJ, Territo PR, Carter GW, Howell GR, Sasner M, Lamb BT (2021). Comprehensive evaluation of the 5XFAD mouse model for preclinical testing applications: a model-ad study. Front Aging Neurosci.

[63] Laurent C, Burnouf S, Ferry B, Batalha VL, Coelho JE, Baqi Y, Malik E, Mariciniak E, Parrot S, Van der Jeugd A, Faivre E, Flaten V, Ledent C, D'Hooge R, Sergeant N, Hamdane M, Humez S, Muller CE, Lopes LV, Buee L, Blum D (2016). A2A adenosine receptor deletion is protective in a mouse model of tauopathy. Mol Psychiatry.

[64] Temido-Ferreira M, Ferreira DG, Batalha VL, Marques-Morgado I, Coelho JE, Pereira P, Gomes R, Pinto A, Carvalho S, Canas PM, Cuvelier L, Buée-Scherrer V, Faivre E, Baqi Y, Müller CE, Pimentel J, Schiffmann SN, Buée L, Bader M, Outeiro TF, Blum D, Cunha RA, Marie H, Pousinha PA, Lopes LV (2020). Age-related shift in LTD is dependent on neuronal adenosine A(2A) receptors interplay with mGluR5 and NMDA receptors. Mol Psychiatry.

[65] Dall'Igna OP, Porciúncula LO, Souza DO, Cunha RA, Lara DR (2003). Neuroprotection by caffeine and adenosine A2A receptor blockade of beta-amyloid neurotoxicity. Br J Pharmacol.

[66] Faivre E, Coelho JE, Zornbach K, Malik E, Baqi Y, Schneider M, Cellai L, Carvalho K, Sebda S, Figeac M, Eddarkaoui S, Caillierez R, Chern Y, Heneka M, Sergeant N, Müller CE, Halle A, Buée L, Lopes LV, Blum D (2018). Beneficial effect of a selective adenosine A2A receptor antagonist in the APPswe/PS1dE9 mouse model of Alzheimer’s disease. Front Mol Neurosci.

[67] Houghton V, Du Preez A, Lefèvre-Arbogast S, de Lucia C, Low DY, Urpi-Sarda M, Ruigrok SR, Altendorfer B, González-Domínguez R, Andres-Lacueva C, Aigner L, Lucassen PJ, Korosi A, Samieri C, Manach C, Thuret S (2020). Caffeine compromises proliferation of human hippocampal progenitor cells. Front Cell Dev Biol.

[68] Han M-E, Park K-H, Baek S-Y, Kim B-S, Kim J-B, Kim H-J, Oh S-O (2007). Inhibitory effects of caffeine on hippocampal neurogenesis and function. Biochem Biophys Res Commun.

[69] Wentz CT, Magavi SSP (2009). Caffeine alters proliferation of neuronal precursors in the adult hippocampus. Neuropharmacology.

[70] Mao Z-F, Ouyang S-H, Zhang Q-Y, Wu Y-P, Wang G-E, Tu L-F, Luo Z, Li W-X, Kurihara H, Li Y-F, He R-R (2020). New insights into the effects of caffeine on adult hippocampal neurogenesis in stressed mice: Inhibition of CORT-induced microglia activation. FASEB J.

[71] Sahu S, Kauser H, Ray K, Kishore K, Kumar S, Panjwani U (2013). Caffeine and modafinil promote adult neuronal cell proliferation during 48h of total sleep deprivation in rat dentate gyrus. Exp Neurol.

[72] Landel V, Baranger K, Virard I, Loriod B, Khrestchatisky M, Rivera S, Benech P, Feron F (2014). Temporal gene profiling of the 5XFAD transgenic mouse model highlights the importance of microglial activation in Alzheimer's disease. Mol Neurodegener.

[73] Atik A, De Matteo R, Boomgardt M, Rees S, Harding R, Cheong J, Rana S, Crossley K, Tolcos M (2019). Impact of high-dose caffeine on the preterm ovine cerebrum and cerebellum. Front Physiol.

[74] Simon R, Brylka H, Schwegler H, Venkataramanappa S, Andratschke J, Wiegreffe C, Liu P, Fuchs E, Jenkins NA, Copeland NG, Birchmeier C, Britsch S (2012). A dual function of Bcl11b/Ctip2 in hippocampal neurogenesis. EMBO J.

[75] Wang H, Warner-Schmidt J, Varela S, Enikolopov G, Greengard P, Flajolet M (2015). Norbin ablation results in defective adult hippocampal neurogenesis and depressive-like behavior in mice. Proc Natl Acad Sci USA.

[76] Cao C, Wang L, Lin X, Mamcarz M, Zhang C, Bai G, Nong J, Sussman S, Arendash G (2011). Caffeine Synergizes with another coffee component to increase plasma GCSF: linkage to cognitive benefits in Alzheimer's mice. J Alzheimers Dis.

[77] Schneider A, Krüger C, Steigleder T, Weber D, Pitzer C, Laage R, Aronowski J, Maurer MH, Gassler N, Mier W, Hasselblatt M, Kollmar R, Schwab S, Sommer C, Bach A, Kuhn H-G, Schäbitz W-R (2005). The hematopoietic factor G-CSF is a neuronal ligand that counteracts programmed cell death and drives neurogenesis. J Clin Invest.

[78] Drapeau E, Mayo W, Aurousseau C, Le Moal M, Piazza PV, Abrous DN (2003). Spatial memory performances of aged rats in the water maze predict levels of hippocampal neurogenesis. Proc Natl Acad Sci USA.

[79] Moser M-B, Moser EI (1998). Functional differentiation in the hippocampus. Hippocampus.

[80] Bannerman DM, Rawlins JNP, McHugh SB, Deacon RMJ, Yee BK, Bast T, Zhang WN, Pothuizen HHJ, Feldon J (2004). Regional dissociations within the hippocampus—memory and anxiety. Neurosci Biobehav Rev.

[81] Fanselow MS, Dong HW (2010). Are the dorsal and ventral hippocampus functionally distinct structures?. Neuron.

[82] Brancati GE, Rawas C, Ghestem A, Bernard C, Ivanov AI (2021). Spatio-temporal heterogeneity in hippocampal metabolism in control and epilepsy conditions. Proc Natl Acad Sci USA.

[83] Debski KJ, Ceglia N, Ghestem A, Ivanov AI, Brancati GE, Bröer S, Bot AM, Müller JA, Schoch S, Becker A, Löscher W, Guye M, Sassone-Corsi P, Lukasiuk K, Baldi P, Bernard C (2020). The circadian dynamics of the hippocampal transcriptome and proteome is altered in experimental temporal lobe epilepsy. Sci Adv.

