# Supplementary material for: Long-term caffeine treatment of Alzheimer mouse models ameliorates behavioural deficits and neuron loss and promotes cellular and molecular markers of neurogenesis
Source: Cell Mol Life Sci. 2021 Dec 16;79(1):55. doi: 10.1007/s00018-021-04062-8 (PMC8738505; doi:10.1007/s00018-021-04062-8)
Supplement: Supplementary file 1 — Supplementary file1 (PDF 729 KB) [file 18_2021_4062_MOESM1_ESM.pdf]

Supplementary information

Martina Stazi, Sandra Lehmann, M. Sadman Sakib, Tonatiuh Pena-Centeno, Luca Büschgens, Andre Fischer, Sascha Weggen, Oliver Wirths

Long-term caffeine treatment of Alzheimer mouse models ameliorates behavioural deficits and neuron loss and promotes cellular and molecular markers of neurogenesis

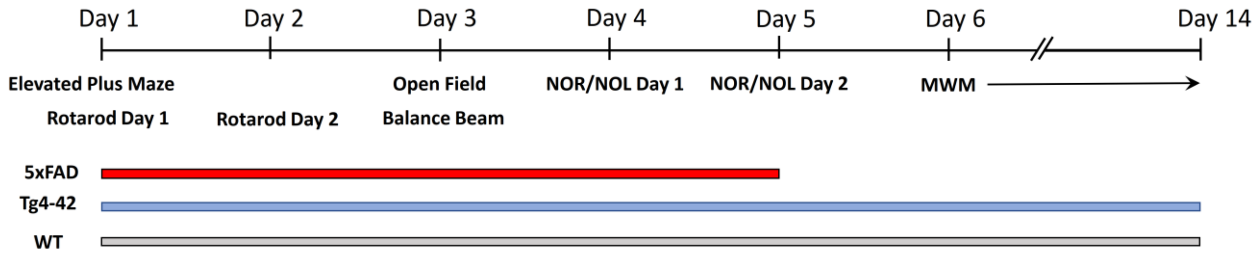

Supplemental Fig. 1: Scheme illustrating the timeline and order of the motor and learning and memory task battery.

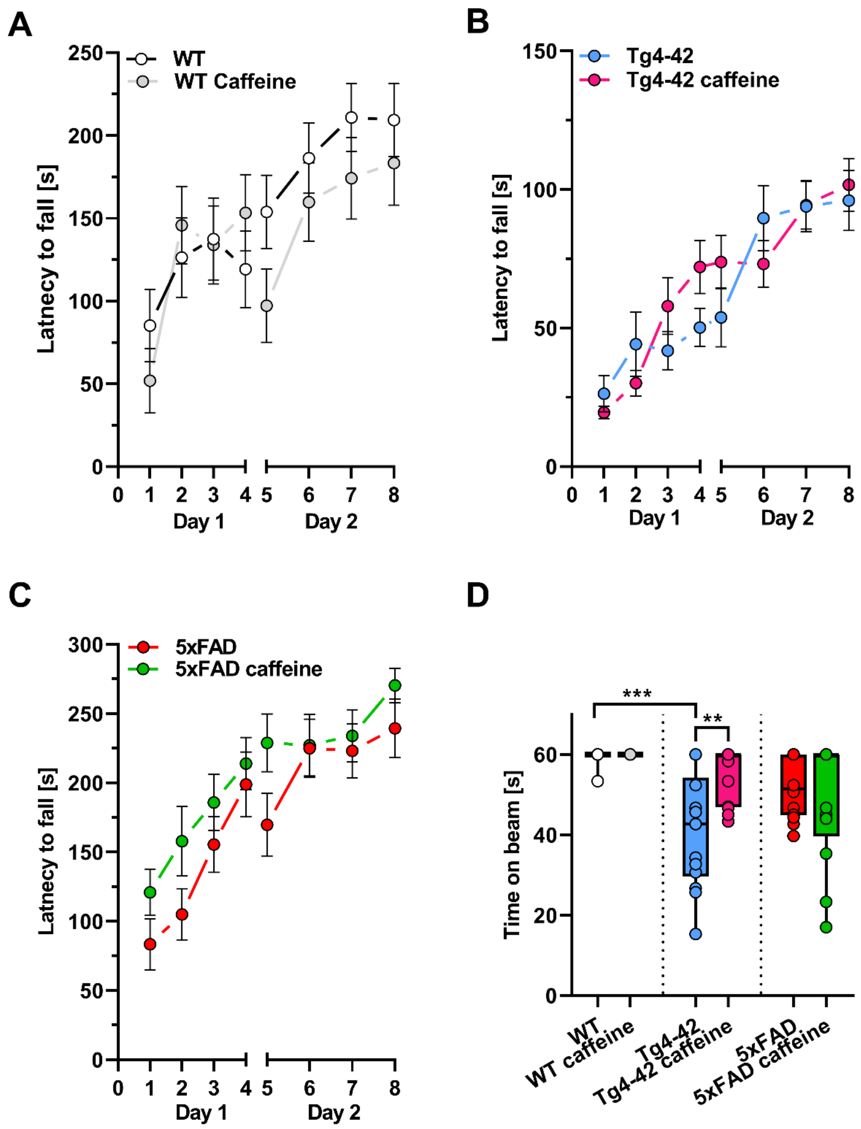

Supplemental Fig. 2: Motor performance in untreated and caffeine-treated WT, Tg4-42 and 5xFAD mice. No changes were detected in motor learning abilities in WT (A), Tg4-42 (B) and 5xFAD mice (C) after caffeine treatment in the rotarod task. In the balance beam test, Tg4-42 performed significantly worse than WT mice, but this phenotype was rescued by caffeine treatment (D) (n = 13-14 per group). (A-C) Two-way repeated measures ANOVA, (D) Mann-Whitney test; $^{*}p < 0.05$. Data are presented as mean ± SEM (A-C) or mean ± SD.

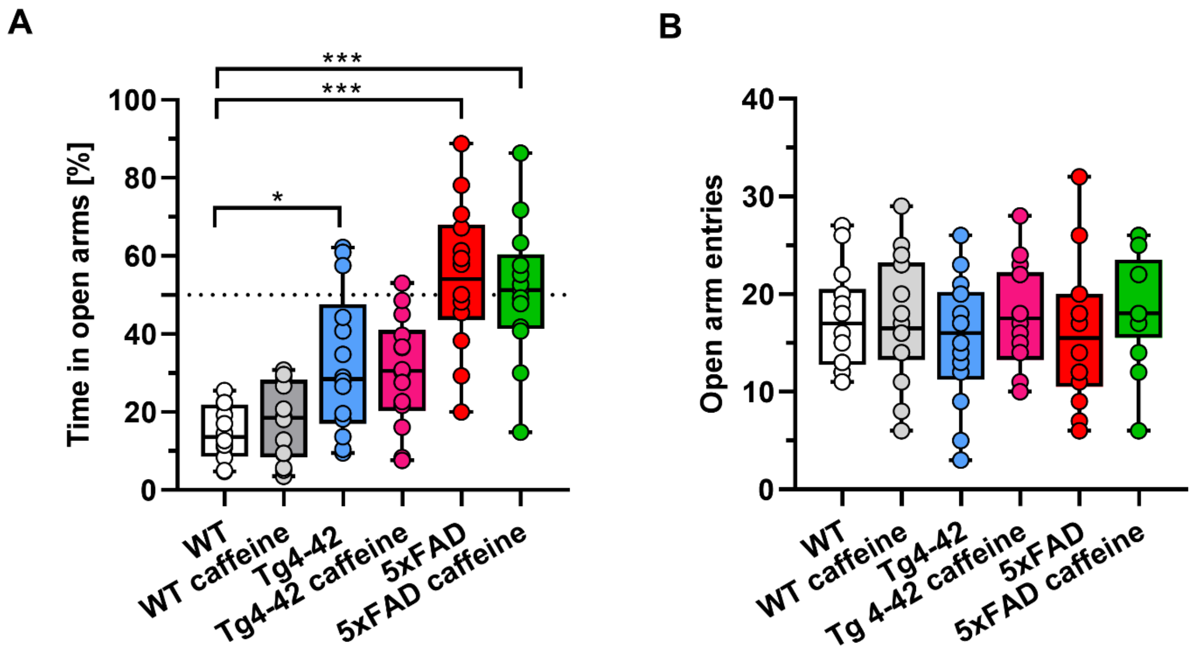

Supplemental Fig. 3: Altered anxiety levels in Tg4-42 and 5xFAD mice. Both Tg4-42 and 5xFAD mice spent an increased amount of time in the open arms of the elevated plus maze (A), but the number of overall open arm entries was not different among all experimental groups (B) (n = 13-14 per group). One-way ANOVA with Tukey's multiple comparison tests; $^{*}p < 0.05$; $^{***}p < 0.001$. Data are presented as means ± SD.

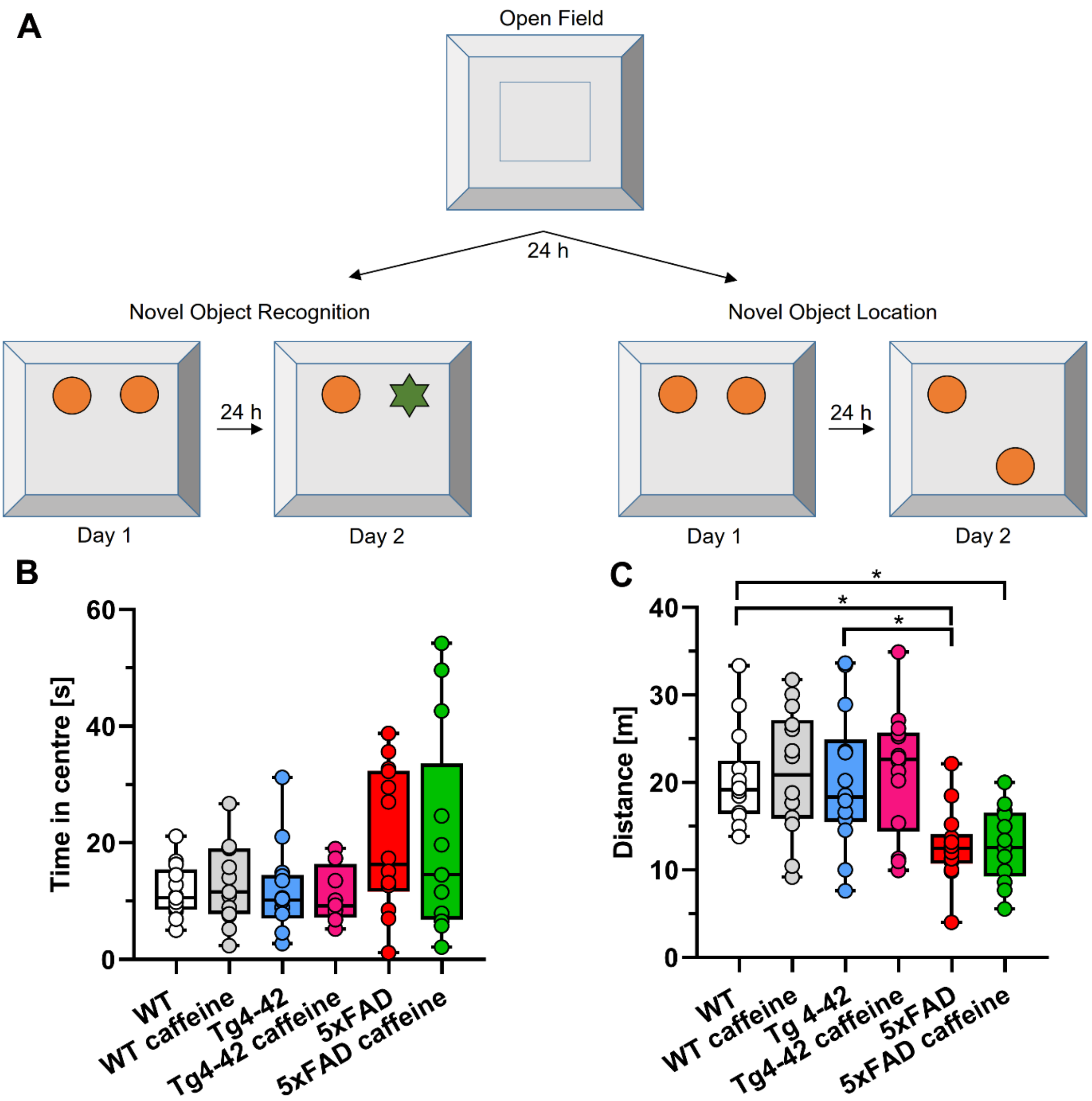

Supplemental Fig. 4: Schematic representation of the open field (OF), novel object recognition (NOR) and novel object location (NOL) tasks (A). Effect of prolonged caffeine treatment on locomotor activity and exploration behaviour in Tg4-42 and 5xFAD mice. (D-G) No significant differences could be detected in the open field task between untreated and caffeine-treated groups with regard to the time spent in the centre (B). Compared to WT and Tg4-42 mice, 5xFAD travelled a reduced overall distance in the OF (C) (n = 12-14 per group). (B, C) One-way ANOVA followed by Bonferroni's multiple comparison tests; $^{*}p < 0.05$. Data are presented as means ± SD.

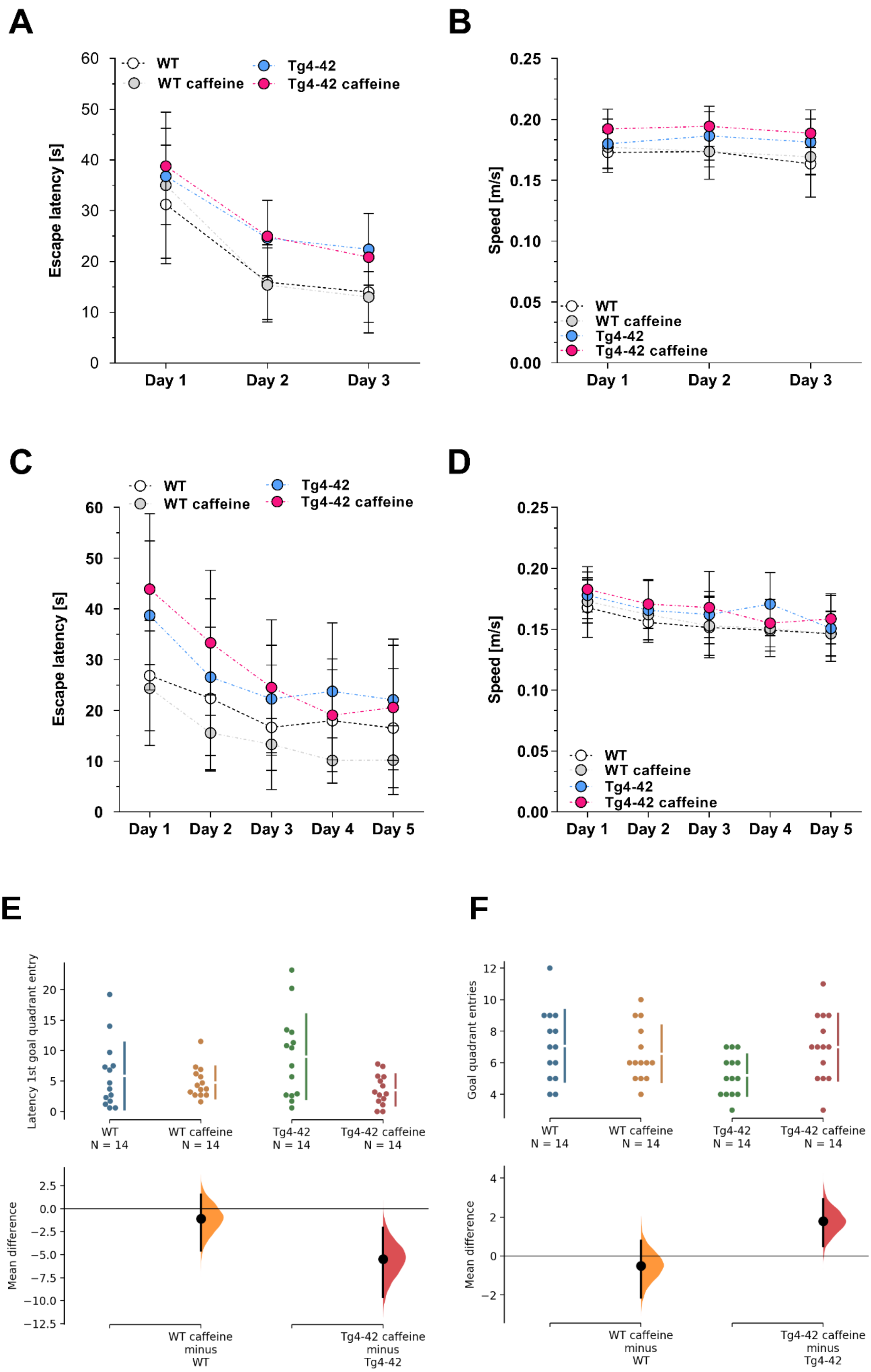

A
Escape latency [s]
WT
WT caffeine
Tg4-42
Tg4-42 caffeine
Day 1
Day 2
Day 3
B
Speed [m/s]
WT
WT caffeine
Tg4-42
Tg4-42 caffeine
Day 1
Day 2
Day 3
C
Escape latency [s]
WT
WT caffeine
Tg4-42
Tg4-42 caffeine
Day 1
Day 2
Day 3
Day 4
Day 5
D
Speed [m/s]
WT
WT caffeine
Tg4-42
Tg4-42 caffeine
Day 1
Day 2
Day 3
Day 4
Day 5
E
Latency 1st goal quadrant entry
WT N = 14
WT caffeine N = 14
Tg4-42 N = 14
Tg4-42 caffeine N = 14
Mean difference
WT caffeine minus WT
Tg4-42 caffeine minus Tg4-42
F
Goal quadrant entries
WT N = 14
WT caffeine N = 14
Tg4-42 N = 14
Tg4-42 caffeine N = 14
Mean difference
WT caffeine minus WT
Tg4-42 caffeine minus Tg4-42

Supplemental Fig. 5: Improved spatial memory performance in Tg4-42 mice after caffeine treatment. All groups showed progressively decreasing escape latencies over the 3 days of cued training (A) and over the 5 days of acquisition training (C). During the cued and acquisition phase, Tg4-42 mice displayed a lower escape latency compared to WT mice (A, C), but swimming speeds were unaltered (B, D). Caffeine-treated Tg4-42 mice showed significantly reduced latency to enter the target quadrant (E) and more target quadrant entries and compared to untreated Tg4-42 mice (F) (n = 14 per group). (A-D) One-way ANOVA followed by Bonferroni's multiple comparison tests. (E, F) Each mean difference in changes in discrimination index score is plotted as a bootstrap sampling distribution. Mean differences are depicted as dots; 95% CIs are indicated by the ends of the vertical error bars. $^{*}p < 0.05$. All data were given as mean ± SD.

Estimation statistics in E and F were done as described in [1]. In brief, for estimation based on confidence intervals (CIs), raw data was directly introduced in https://www.estimationstats.com/ [2] and the results and graphs were downloaded. Cumming estimation plots show the mean difference for two comparisons. The raw data are plotted on the upper axes. Gapped lines show summary measurements (mean ± SD) for each group. Each mean difference is plotted on the lower axes as a bootstrap sampling distribution. Five thousand bootstrap samples were taken; the CI was bias-corrected and accelerated. Mean differences are depicted as dots; 95% CIs are indicated by the ends of the vertical error bars. To measure the effect size, we used unbiased Cohen's *d* (also known as standardized mean difference).

References

1. Manouze H, Ghestem A, Poillerat V, Bennis M, Ba-M'hamed S, Benoliel JJ, Becker C, Bernard C (2019) Effects of Single Cage Housing on Stress, Cognitive, and Seizure Parameters in the Rat and Mouse Pilocarpine Models of Epilepsy. eneuro 6 (4):ENEURO.0179-0118.2019. doi:10.1523/eneuro.0179-18.2019
2. Ho J, Tumkaya T, Aryal S, Choi H, Claridge-Chang A (2019) Moving beyond P values: data analysis with estimation graphics. Nature Methods 16 (7):565-566. doi:10.1038/s41592-019-0470-3
